# Actions of metformin and statins on lipid and glucose metabolism and possible benefit of combination therapy

**DOI:** 10.1186/s12933-018-0738-4

**Published:** 2018-06-30

**Authors:** Mariël F. van Stee, Albert A. de Graaf, Albert K. Groen

**Affiliations:** 1 Netherlands Organisation for Applied Scientific Research (TNO), Zeist, The Netherlands; 2 Amsterdam Diabetes Center and Department of Vascular Medicine, Academic Medical Center, Amsterdam, The Netherlands; 3 Department of Laboratory Medicine, University of Groningen, University Medical Center Groningen, Groningen, The Netherlands

**Keywords:** Type 2 diabetes, Metformin, Atorvastatin, Simvastatin, Combination therapy, Dyslipidemia, Glucose metabolism, Lipid metabolism

## Abstract

Patients with diabetes type 2 have an increased risk for cardiovascular disease and commonly use combination therapy consisting of the anti-diabetic drug metformin and a cholesterol-lowering statin. However, both drugs act on glucose and lipid metabolism which could lead to adverse effects when used in combination as compared to monotherapy. In this review, the proposed molecular mechanisms of action of statin and metformin therapy in patients with diabetes and dyslipidemia are critically assessed, and a hypothesis for mechanisms underlying interactions between these drugs in combination therapy is developed.

## Background

Type 2 diabetes mellitus (T2DM) and cardiovascular diseases are common medical conditions that are often found comorbid with each other. T2DM is a metabolic disorder characterized by increased plasma glucose concentration (hyperglycemia) caused by persistent insulin resistance, and progressive β cell failure. Insulin resistance is a normal reaction of the body to cope with an excess of circulating triglycerides. Normally, food intake causes only a transient insulin resistance, but currently the excess of energy intake in humans in many regions of the world is almost constant causing sustained insulin resistance leading to T2DM often in combination with dyslipidemia.

Metformin is used as first line treatment of T2DM due to its primary pharmacological effect of controlling disturbed glucose metabolism [1]. Interestingly, despite its longstanding and widespread use, the molecular mechanism via which the drug acts is still a matter of considerable controversy as it additionally influences lipid/cholesterol pathways. Dyslipidemia (or diabetic dyslipidemia when diagnosed in T2DM patients) is an abnormality in lipid metabolism; i.e. quantitatively observed in abnormalities in blood lipids including increased triglycerides (TG) and low-density lipoprotein (LDL-C) and a decreased high-density lipoprotein cholesterol (HDL-C) concentration [2]. These disturbances are established risk factors in the development of atherosclerosis [3]. Metformin treatment double dose (500–750 mg/day) given to T2DM patients showed after 12 weeks a decreased central blood pressure suggesting a beneficial influence on cardiovascular disease [4]. Table 1 (Supplementary) shows recent articles related to diabetic dyslipidemia. Statins are prescribed as first choice treatment for (T2DM) patients with dyslipidemia, because of their impressive LDL-C lowering effects. Dyslipidemia and insulin resistance are potential risk factors for myocardial infarction [5]. Therefore, it is also important to initiate statin therapy in diabetic patients without known cardiovascular disease [5]. Paradoxically long-term statin therapy when given as monotherapy to dyslipidemic patients is associated with increased incidence of T2DM [6, 7]. Both metformin and statins thus act on glucose—as well as lipid metabolism which is why metformin–statin combination therapy is prescribed to many T2DM patients. However, the therapeutic effects of different doses of statin and metformin given in combination have not been thoroughly and systematically investigated. Since both drugs act on glucose as well as lipid metabolism, it is important to understand in detail the interactions between metformin and statin action on metabolism to be able to design treatments with optimal safety/efficacy profiles

**Table 1:** Overview of areas and focus of recent review articles about diabetes dyslipidemia and the corresponding focus area for each review is given

Area	Focus	References
Pathophysiology	No specific focus/general	Besseling and Hutten [153], Cederberg et al. [154], Checade et al. [155], Feingold and Grunfeld [156], Jaiswal et al. [157], Ng [158], Soran et al. [159], Schofield et al. [160], Verges[161], Wang et al. [162], Wu and Parhofer [2]
	Intestine	Arca [163], Tomkin and Owens [164]
	Liver	Arca et al. [165], Perry et al. [166], Taskinen and Boren [167]
Relation IR and lipids	–	Pagidipati et al. [168], Parhofer [169], Patel et al. [170], Perry et al. [166]
CV risk	–	Wang et al. [162]
Pharmacotherapy/treatment	Treatment of dyslipidemia	Cederberg et al. [154], Checade et al. [155], Dake and Sora [171], Halcox and Misra [172], Ng [158], Paneni and Cosentino [173], Schofield et al. [160], Szalat et al. [174], Wu and Parhofer [2]
	Effects drugs on lipid and glucose metabolism	Ferrannini and DeFronzo [175], Soran et al. [159]
Complications	–	Balakumar [176], Chen and Tseng [177], Leon and Maddox [178], Paneni et al. [179]

The aim of this article is therefore to provide insight in the molecular mechanism of statin/metformin interaction which can help to determine an optimal dosing strategy of both drugs.

## Antidiabetic drug metformin and the potential mechanisms by which it may affect lipid metabolism

Metformin, discovered in 1922, came on the market as late as 1979 [8]. It belongs to the biguanide class of drugs, and is a derivative from guanidine found in *Galega officinalis*. Metformin is available in different formulations including immediate-release metformin, extended-release metformin [9], and delayed-release metformin [10]. The latter two forms were developed to expand the absorption of metformin along the gut (Table 2). Administration of metformin 30 min before a meal showed highest therapeutic efficacy in lowering postprandial hyperglycemia [11].

**Table 2:** Different formulations and the corresponding relevant characteristics of the oral drug metformin (information from [180])

Metformin formulation	Dose	C_max_ (ng/ml)	t_max_ (h)	AUC_mean_ (ng*h/ml)	Properties	Refs
Immediate release (IR)	1000 mg bid	1328	3.5	18,710	Rapid gut absorption 90% absorption within 30 min High systemic exposure GI side effects High systemic exposure	[180]
Extended release (ER) [181]	2000 mg qd	1688	5.1	16,990	Two-phase approach tablet using polymers Prolonged gut absorption (duodenum and jejunum) 90% absorption within 10 h	[9, 182]
Delayed release (DR) [183]	500 mg bid/1000 mg bid	905/607	9/9	9010/6160	Enteric coated (polymers) core tablet Release of metformin by pH of 6.5 (distal small intestine) Late gut absorption (ileum) Increased GLP-1 secretion Less systemic exposure	[10]

*qd* once a day, *bid* twice a day

### Chemical characteristics

Metformin hydrochloride has a molecular weight of 165.6 g/mol and contains 4 hydrogen bond donors and 1 hydrogen bond acceptor. There is only a limited understanding of the physicochemical properties of metformin in solvent [12]. Metformin hydrochloride shows two distinct pKa values referring to different protonated forms of metformin (Table 3). The pKa values were reported as 2.8 and 11.6 [13], but using a more accurate pH determination as 3.1 and 13.8 [14].

**Table 3:** Percentage of fractional ionization of metformin after oral administration in different organs/tissues calculated using the Henderson–Hasselbalch equation

Human body	pH	Metformin chemical form			Absorption
		[B]	[HB^+^]	[H_2_B^2+^]	
		
Oral cavity	7	0	99.99	0.01	–
Stomach	2	0	7.36	92.64	10%
Jejunum + ileum	8	0	100	0	60%
Duodenum	6.25	0	99.93	0.07	20%
Plasma/liver	7.4	0	99.99	0.01	–

The pH in the different organs/tissues/plasma leads to different forms of metformin. Neutral metformin, which is a base, will only be dominant at very high pH. The monoprotonated conversion of metformin with a stabilized cation (equally distribution between the nitrogen atoms) occurs in a neutral environment (pH ≈ 7). Biprotonation of metformin appears by a decreasing pH value

### Intracellular localization

As is evident from Table 3, metformin is present for over 99% in the monoprotonated form in all tissues of the body except the stomach. The charge on the molecule precludes passive diffusion across lipid bilayers. To be able to evaluate the interaction of metformin with its putative targets it is important to know the intracellular and intraorganellar concentration. However, these data are difficult to obtain because of the difficulties in subfractionation of the cell in its different compartments (cytoplasm, mitochondria, nucleus, etc.). The sparse data reported, indicate that metformin is mostly distributed in the cytosolic fraction (~ 70%) of rat hepatocytes compared to mixed membranes (12%), nucleus (~ 5%), and mitochondrial and lysosomal fractions (8%) [15]. A low binding affinity of metformin to mitochondrial membranes was seen, perhaps because of the two methyl groups of metformin [15], but perhaps also due to the used fractionation technique [16]. However, the mitochondrial membrane potential may promote entry to the positively charged metformin [17], which will then concentrate inside the negatively charged mitochondria [18]. Modelling of the metformin distribution and validation confirmed the presence of high concentrations of the drug in the mitochondria and the endoplasmic reticulum (ER), dependent on the membrane potential [19].

### Distribution of metformin concentrations over organs, tissues, and plasma

Metformin is able to (in)directly interact with many enzymes [e.g. mitochondrial electron transport chain complex I, AMPK, glycerol 3-phosphate dehydrogenase (mGPD)], which lead to a large diversity of possible effects of the drug. When scrutinizing literature on metformin effects, one needs to consider that in many studies supraphysiological concentrations of metformin were used [20]. The reported cellular sites of actions and effects thus may not reflect the in vivo situation when metformin is given to T2DM patients. Normally, the therapeutic window of metformin in plasma is between 1 and 50 μM [21]. Table 4 presents an overview of metformin concentrations as observed in vivo in organs/tissues of humans, mice, and rats upon administration of doses that lie in the range of therapeutic applications in humans. Accumulation of metformin occurs majorly in the intestine, but also in the stomach, liver, kidney and to a lesser extent in muscle. The accumulation of metformin in intestine and stomach is not surprising in view of the fact that these organs are most exposed to high concentrations of metformin. A recent radiotracer study confirmed the high metformin levels in these organs [22]. These concentrations are at least tenfold higher than metformin concentrations in the liver, indicating that the intestine is probably an important site of action. In fact, the effects of metformin in the intestine may be rather different than the effects in the liver.

**Table 4:** Metformin concentrations in organs/tissues of different organisms after treatment

Organism	Dose	Time (h)	Intestine (μmol/kg wet weight)	Plasma (μmol/l)	Liver (μmol/kg wet weight)	Muscle (μmol/kg wet weight)	Adipose tissue (μmol/kg wet weight)	Stomach (μmol/kg wet weight)	Kidney (μmol/kg wet weight)	Refs
Human	2*850 mg; 6–8 weeks	3	4000	8–24						[30]
Human	2*850 mg; 6–8 weeks	12–16	250	< 8						[30]
Mice	1.25–1.5 mg (50 mg/kg)	0.5, 1, 2, 4, 8	3729; 5554; 3212; 2061; 803;	IVC: 28.9; 28.1; 12.6; 14.7; 6.6 HPV: 51.7; 39; 19; 18.5; 9.3;	182, 154, 115, 37, 32	22, 28, 87, 58, 15	12, 14, 16, 11, 2	800, 965, 331, 129, 124	428, 246, 172, 121, 71	[184]
STZ diabetic mice	1.25–1.5 mg (50 mg/kg)	0.5, 1, 2, 4, 8	2161; 2585; 5546; 1261; 500	IVC: 35.4; 34.3; 21.5; 11.2 HPV: 61.5; 54.9; 28; 14.4; 8.9;	282, 137, 86, 53,32	66, 24, 16, 14, 16	42, 26, 75, 30, 20	541, 367, 783, 64, 52	384, 428, 229, 107, 63	[184]
Rat	~6 mg (50 mg/kg)	0.5		IVC: 14	49.6 ± 13.5					[15]
Rat	~6 mg (50 mg/kg)	4		IVC: 6.5	28.3 ± 7.3					[15]

*IVC* inferior vena cava, *HPV* hepatic portal vein, *STZ* streptozotocin

For the purpose of this review, we will concentrate on the organs considered to be most relevant for interaction of metformin with glucose and lipid metabolism, i.e. the intestine because of the high metformin concentrations, the liver because of the organ’s central role in linking glucose and lipid metabolism, and the pancreatic β cells which secrete insulin and play a key role in the regulation of glucose metabolism. Since metformin concentrations differ between intestine, liver and β cells, different organ-specific effects may be expected.

### Intestinal absorption

After oral administration, metformin is transported in the small intestine across the apical membrane into the enterocytes via several transporter proteins, of which the plasma monoamine transporter (PMAT; SLC29A4), organic cation transporter 1 (OCT1; SLC22A1) and serotonin transporter (SERT; SLC6A4) are considered to be the most likely candidates [23, 24]. Metformin is also a substrate for the thiamine transporter THTR-2 (SLC19A3), with a K_m_ value of 1.15 mM [25]. This binding affinity is comparable with the K_m_ of PMAT (1.32 mM) for metformin [26]. THTR-2 is highly expressed in the intestine, and primarily transports vitamin B1. Polymorphism in the human OCT1 gene has been reported and could cause a reduced transport of metformin leading to the development of metformin intolerance [27]. In Caco-2 cells there is no evidence for a role of apical organic cation transporter 3 (OCT3; SLC22A3) in the uptake of metformin [23]. Transporters mediating metformin efflux from the enterocytes have not been unequivocally identified. The OCT1 has been suggested, but it is not clear whether this transporter could have a dual localization both at the apical and basolateral membrane of the enterocytes [20, 28]. The recently published “sponge” hypothesis, based on experiments on caco-2 cells, suggests that there is no carrier mediated transport to the bloodstream. Rather, metformin accumulates in the cell until the luminal concentration is less than the concentration in the enterocytes, whereupon-it is pumped/leaking out back into the lumen and will travel via paracellular transport to the blood or will be taken up again by cells [29]. This hypothesis has been partly confirmed in experiments with C^11^-labeled metformin followed by positron emission tomography. These experiments revealed a low capacity of basolateral membrane transport activity in humans resulting indeed in accumulation in the cell when there was a higher concentration of metformin in the lumen compared to in the cell [22].

### Mechanisms of metformin action in the intestine

The concentration of metformin in human jejunum has been shown to be 30- to 300-fold greater than in plasma (Table 4), again demonstrating accumulation of metformin in the intestinal mucosa [30]. Because of this remarkably high concentration it seems logical to assume that all known metformin targets present in enterocytes are addressed.

To simplify, we grouped the resulting effects into: decreased lipoprotein synthesis, neural reduction of endogenous glucose production, and increased glucagon-like peptide-1 (GLP-1) production.

#### Decreased lipoprotein synthesis

**Fig. 1:**
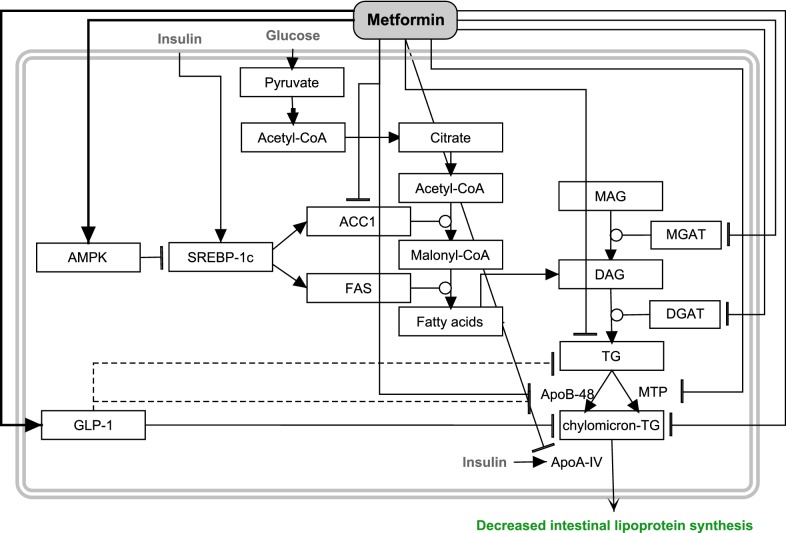
Summary of the effects of metformin on intestinal lipoprotein synthesis in different experimental studies. Key events are the positive regulation of AMPK and GLP-1, from where a spectrum of changes in the fatty acid and TG synthesis, and chylomicron production are observed that effectively result in decreased intestinal lipoprotein synthesis. Arrows represent stimulation, and T-shaped symbols represent inhibition

Dysregulation of intestinal lipoprotein metabolism is often seen in T2DM patients [31–33]. The high concentrations of insulin found in T2DM patients may be responsible for this phenomenon [34]. Lipogenic gene expression, involved in de novo lipogenesis, is regulated by the sterol regulatory element-binding protein-1c (SREBP-1c), which is highly expressed in the upper villus of the jejunum and ileum [35]. SREBP-1c in the intestine is positively regulated by insulin and negatively by AMPK, and is able to upregulate enzymes, such as acetyl-CoA carboxylase (ACC1) and fatty acid synthase (FAS), which are involved in *denovo* fatty acid synthesis [36, 37]. Metformin (2300 mg/day) is able to decrease intestine-derived TG-rich lipoproteins measured in plasma (~ − 50% chylomicrons and ~ − 20% chylomicron remnant lipoprotein fractions) in T2DM patients [34]. Figure 1 shows the possible cellular targets of metformin in enterocytes based on the literature discussed here. Metformin treatment of morbidly obese T2DM patients induced a small decrease in mRNA expression of SREBP-1c, ACC1, and apo A-IV (involved in the secretion of chylomicrons), leading to a slightly improved intestinal lipid homeostasis [33]. Insulin positively regulates synthesis of intestinal apo A-IV as well as secretion [38], which may cause an increase of this protein at the high insulin levels in the presence of insulin resistance. Experiments in animal models confirmed the possible interaction of metformin with proteins and enzymes involved in triglyceride and apo B synthesis.

In addition to the effects discussed above, a decreased chylomicron concentration could also be caused by an elevated GLP-1 concentration in the intestine (induced by metformin; see “Increased GLP-1 production”) by reducing apo B-48, the triglyceride availability, and chylomicron secretion [39, 40], although this is not considered in [33–35, 41].

Summarizing, metformin treatment impacts importantly on lipoprotein synthesis in the intestine, but the molecular mechanism is not yet fully clear and requires further investigation.

#### Neuronal reduction of EGP

The liver and kidneys are the major organs responsible for gluconeogenesis from amino acids or glycerol. However, pioneering work of the group of Mithieux showed that also the human small intestine expresses considerable activity of the gluconeogenic gene [glucose 6-phosphatase (G6Pase)], and may therefore contribute to endogenous glucose production (EGP) [42]. Intestinal gluconeogenesis may play a role in the gut-brain axis. In the post absorptive state, amino acids from the meal are used as a substrate for intestinal gluconeogenesis. This glucose enters the portal vein, where it binds to the sodium-glucose cotransporter 3 (SGLT3). This transporter is activated and signals to the hypothalamus in the brain which indicates the liver to decrease hepatic glucose production [43]. Basal intestinal gluconeogenesis may contribute 5–7% (standard meal) or 20–25% (protein-enriched meal) of total EGP [44] in mice. Whether these estimates can be translated to the human situation is not clear yet.

**Fig. 2:**
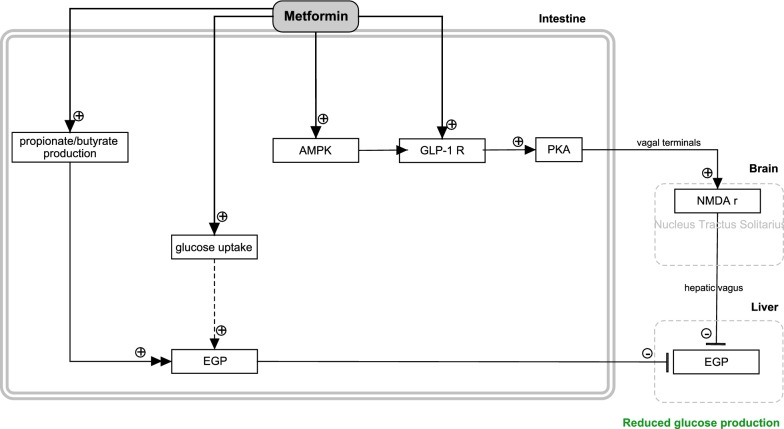
Summary of the effects of metformin in the intestine (small intestine and duodenum) that cause glucose-lowering effects by reducing the hepatic EGP

The question arises whether metformin interacts with intestinal gluconeogenesis. Activities of key enzymes [e.g. phosphoenolpyruvate carboxykinase **(**PEPCK), hexokinase] contributing to small intestine glucose metabolism were not affected during one-week metformin treatment (50 mg/kg/day) in high-fat fed rats [45]. Yet, intestinal glucose uptake and intestinal glucose release were increased, and the overall endogenous glucose production was decreased compared to controls (Fig. 2). Another study showed that metformin treatment induces alterations in the gut microbiome in T2DM patients [46]. This has been reported to increase butyrate and propionate production, possibly via modification of the host lipid absorption [46, 47], which may then support intestinal gluconeogenesis [46].

The last few years, the small intestine has come into view as the prime target of metformin. Experiments with infusion of the drug in the proximal small intestine (50 or 200 mg/kg; 50 min), of obese and diabetic rats [48] indicated that the hepatic glucose production decreased via an AMPK-GLP-1 receptor (GLPR)-protein kinase A (PKA)-dependent neuronal route. The heterotrimer AMPK is a serine/threonine kinase constituted from a catalytic α-subunit and regulatory β- and γ-subunits. The protein plays and important regulatory role in cellular energy metabolism [49]. Several pharmacological compounds (e.g. metformin), natural compounds (rooibos, berberine), hormones (adiponectin, leptin, IL-6), and physiological processes (exercise, fasting, caloric restriction) activate AMPK activity [50]. Activated AMPK has pleiotropic effects on energy metabolism improving insulin resistance and diabetes type 2 [51]. In the proposed intestinal-neuronal pathway, metformin activated intestinal AMPK which interacts with the GLPR and PKA, leading to stimulation of a neuronal signal via the *nervus vagus* to the brain. According to this hypothetical route in the brain, the *N*-methyl-d-aspartate (NMDA) receptors located in the nucleus tractus solitarius (contribute to autonomic regulation) receive this neuronal signal, and react by sending a signal via the hepatic vagus to the liver where it decreases hepatic EGP. Additionally, it was hypothesised that metformins glucose lowering effects on short term (i.e. first drop in glucose concentration after a meal) might be related to intestinal processes, while long-term effects might be dedicated to hepatic processes [48].

#### Increased GLP-1 production

**Fig. 3:**
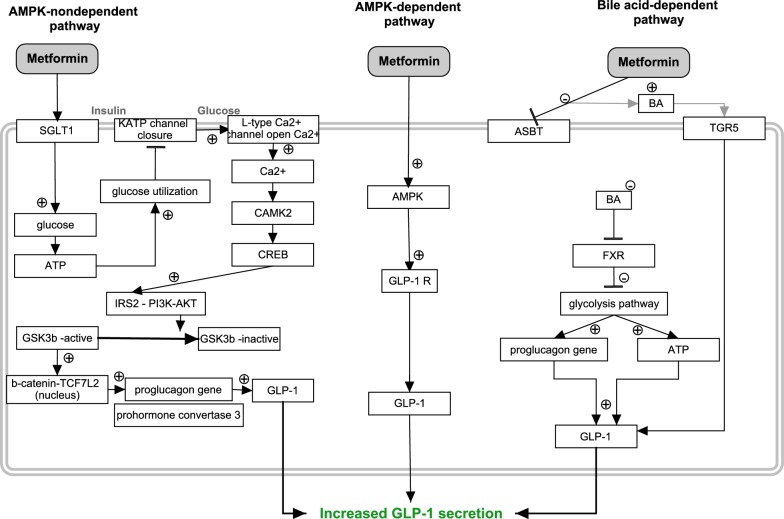
Summary of the effects of metformin discussed in the text that cause increased intestinal GLP-1 secretion

Production of GLP-1 occurs mainly in the enteroendocrine L cells located mostly in the distal part of the small intestine and in the colon, while it can also be released by α-cells from the pancreas [52]. The regulation of GLP-1 production is complex and involves a combination of nutrient, hormonal and neural stimuli [53]. Increased fasting total and active GLP-1 as well as circulating total GLP-1 concentrations have been measured in obese (T2DM) patients on metformin treatment [54–56], Different mechanisms have been proposed to explain this increase [57]. An AMPK-dependent pathway, an AMPK-independent pathway, and a bile acid mediated pathway, have been proposed to explain the effects of metformin on GLP-1 secretion (Fig. 3) as discussed below.

Several studies have suggested mechanisms responsible for the increased GLP-1 secretion observed during metformin treatment in which AMPK plays a prominent role [48]. In the human small intestine, AMPK is present in the apical part of the small intestine, mainly at the lumen villus absorptive cells (i.e. below brush border and in stromal cells) and fulfils important functions in metabolic pathways, leading to favourable effects during metformin treatment [41]. Whereas in rats on metformin treatment (50 or 200 mg/kg) the need of duodenal AMPK in order to activate GLPR was confirmed [48].

##### Non-AMPK dependent pathways

Other non-AMPK dependent pathways exist, for instance, it is argued in [58] that effects on GLP-1 production by metformin (1 mM in NCI-H716 human intestinal cells and/or 12.5 mg/kg body weight in mice) were not mediated through AMPK signalling. Instead, [58] demonstrated that increased expression of precursors of GLP-1 on L-cells is regulated through increased glucose entering in the cell via SGLT1 located in the brush border membrane of the lumen. Increased glucose uptake by SGLT1 was a consequence of an increased expression of SGLT1. Genes involved in glycolysis/TCA cycle/electron regulation cause closing of the K^+^ channel and opening of the Ca^2+^ channel. The increased Ca^2+^ flux activates the calcium–calmodulin dependent kinase 2 (CAMK2) and cAMP response element binding protein (CREB) resulting in the activation of the insulin signalling pathway [insulin receptor substrate 2 (IRS2), phosphoinositide 3 kinase (PI3K), and protein kinase B (AKT)], and consequently inhibition of glycogen synthase kinase 3 (GSK-3b). This causes b-catenin, a protein involved in transduction of signals to the nucleus, to move to the nucleus where it merges with transcription factor 7-like 2 (TCF4L2), leading to an increased proglucagon activity (a precursor of GLP-1), and GLP-1 production [58]. However, in a different study only a slightly increased expression of b-catenin and no effect on the expression of TCF7L2 was observed in the nucleus of NCI-H716 human intestinal cells during metformin treatment (1 mM) [59]. The precursors of GLP-1, proglucagon and prohormone convertase 3 were also upregulated in this study, causing elevated secretion of GLP-1 [59]. GLPR on the afferent vagus nerve triggers a gut-brain-liver network, which may decrease the hepatic glucose production [48].

Metformin might also indirectly act on GLP-1 secretion via the modulation of bile acids in the intestine, for which [55] summarized two potential mechanisms. Firstly, metformin inhibits the intestinal apical sodium-dependent bile acid transporter (ASBT), causing bile acids (BA) to accumulate in the intestinal lumen. Therefore, the apical G protein-coupled bile acid receptor 1 (TGR5) is stimulated which will cause an increased secretion of GLP-1. Secondly, because of the inhibition of ASBT the concentration of BA in the illeoocyte will decrease, resulting in decreased activation of the nuclear farnesoid X receptor (FXR). Lack of FXR activation results in inhibition of the glycolytic pathway and activates the expression of proglucagon and intracellular ATP, leading to increased GLP-1 production and secretion [60].

##### Metformin decreases bile acid concentration in enterocytes and modulates the gut microbiota

The concentration of bile acids in the intestine is the result of multiple processes involved in the enterohepatic circulation (~ 6 times daily) that consists of: release of bile by the hepatocytes into the bile canaliculi, the storage of bile in the gall bladder where it is concentrated, release of bile after consuming a meal, flux of bile into the duodenum, the reabsorption of bile acids by the sodium-dependent intestinal ASBT, passive diffusion mechanisms to the portal vein, and the re-uptake by hepatocytes.

Hepatobiliary transport (transport from the sinusoid to the bile) of metformin in humans, rats and mice, is negligible indicating that uptake of metformin in the small intestine occurs only by a first pass mechanism [22, 61, 62]. Metformin (> 1000 mg/day) is able to inhibit bile acid absorption in the intestine, leading to a decreased serum bile acid concentration [55] and an increased faecal bile acid excretion in T2DM patients [63]. The altered metabolism of bile acids by metformin may also be the reason why metformin influences the composition of the gut microbiome. This has developed into an important subject of research after high throughput sequencing platforms became available, and altered metabolic functions/composition of the microbiota were observed in obese/diabetic patients [64]. The gut microbiome composition is associated with dyslipidemia and insulin resistance [65]. Since the gut bacteria are important in bile acid metabolism and thereby may influence host metabolism via the nuclear hormone receptor FXR and TGR5 signalling pathways [66] part of the metformin effects on host metabolism may be secondary via this route. Recently, the effects of metformin on the gut microbiota composition in T2DM patients were investigated [67]. The composition was changed, including an increased abundance of *Akkermansia muciniphila* (related to metabolic health [68]) and multiple bacteria involved in the short-chain fatty acid (i.e. butyrate, propionate) production. De la Cuesta-Zuluaga et al. suggest that the improved metabolic health is associated with a stronger intestinal mucosal barrier caused by the affected bacteria [67]. Diversity of the microbiota may also contribute to the different observations seen in T2DM treated with metformin [69]. The effect of metformin on gut microbiota composition was confirmed in a recent randomised controlled trial in T2DM patients [47]. Transplantation of fecal microbiota derived from metformin-treated subjects to germ-free mice improved glucose tolerance compared to mice that received fecal microbiota from placebo-treated controls. This indicates that changes in gut microbiota induced by metformin treatment mediate part of the beneficial effects of this drug on glucose homeostasis [47]. Alterations in bile acid metabolism may partly explain the effects.

### Mechanisms of metformins action in the liver

**Fig. 4:**
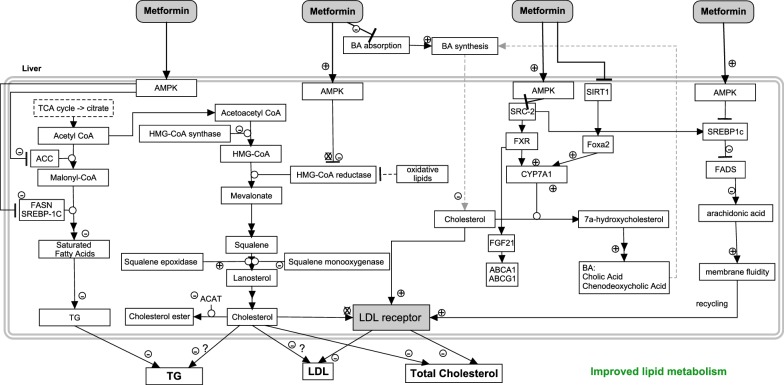
Summary of the effects of metformin in the liver that cause an overall improved lipid metabolism by reducing triglycerides, LDL-C, and total cholesterol

Metformin navigates to the liver via the portal vein and is taken up predominantly by OCT1 [70] as well as by THTR-2 [25]. The main mechanisms of metformin involved in decreasing the endogenous glucose production and plasma glucose have all been extensively and critically reviewed elsewhere [71–73]. In this review, the effects of metformin on the lipid metabolism are highlighted, thereby creating a special focus on the effects on lipids related to the activation of AMPK by metformin. Figure 4 shows the specific interactions of metformin resulting in an improved lipid metabolism.

#### AMPK-dependent metformin effects on HMG-CoA reductase

Metformin activated AMPK is able to modulate cholesterol synthesis as well. Phosphorylation of 3-hydroxy-3-methyl-glutaryl-coenzyme A reductase (HMGCR) will decrease cholesterol biosynthesis [74]. Besides the phosphorylation of the protein downregulated mRNA levels (≈ 70%) of HMGCR and HMG-CoA synthase (HMGCS) were detected in rat FaO hepatoma cells treated with 2*10^−3^ and 5*10^−3^ M metformin [75]. This drastic decrease was, however, not observed when metformin was administered in a lower more physiological relevant concentration (1*10^−3^ M). Treatment of rat primary hepatocytes with metformin (0.5–5*10^−4^ M) induced inhibition of HMGCR and HMGCS mRNA expression [76]. A not significant decrease of HMGCR activity of human cultured fibroblast was observed with metformin treatment (4226 ± 413, no metformin vs. 4082 ± 396 pmol/h per mg, 5*10^−5^ M metformin) [77]. In another study, the cholesterol biosynthesis rate from [^3^H] acetate, but not from [^14^C] mevalonate, was suppressed by 11% following murine macrophage cell incubation with 2*10^−3^ M metformin, respectively [78]. This indicates that metformin is able to slightly inhibit macrophage HMGCR, even though relatively high concentrations were chosen. Metformin treatment of mice decreased both triglyceride (~ − 35%) and LDL-C (~ − 30%) concentrations in plasma [79]. Interestingly, a study in 1983 showed the differences of HMGCR in the liver and intestine of diabetic rats before and after 250 mg/kg metformin treatment [80]. Hepatic HMGCR was not affected by metformin treatment, while intestinal HMGCR showed a decrease in activity of ~ 62%. The Acyl-CoA cholesterol acyltransferase (ACAT) involved in the catalysis of cholesterol esters showed as well decreased activity (~ − 35%). This cholesterol lowering effect in the intestine may lead to beneficial effects on cholesterol metabolism, and further supports the hypothesis that the intestine is an important target organ of metformin. In a nutshell, metformins action on HMGCR is weak in hepatocytes, and it is plausible that other pathways are involved in achieving the lipid lowering effects of metformin.

#### Other mechanisms of metformin effecting the lipid metabolism

Metformin shows beneficial effects on the glucose and lipid metabolism [81], even though the pathways and the corresponding strengths are not fully understood. Part of the variation in metformin efficacy may be due to the presence of responders and non-responders to metformin treatment [82, 83], racial and ethnic background [84], and personal variation in the adaptation of metformin treatment. In the literature, different pathways are suggested that could contribute to the positive effects of the drug the lipid metabolism (Fig. 4).

A pathway inducing reduction of LDL cholesterol has been proposed by Sonne et al. [85] and is as follows. Inhibition of the intestinal absorption of bile acids by metformin causes an increased synthesis of bile acids in the liver, and cholesterol is used for this process [86], thereby causing a decreased amount of cholesterol in the hepatocytes. Upregulation of the LDL-C receptor may increase the uptake of lipoproteins, to restore a sufficient level of cholesterol in the liver. Hereby, the LDL-C concentration and plasma total cholesterol concentrations may indirectly decrease by the action of metformin. However, it should be noted that this mechanism could account for only marginal effects. Major increases in bile acid excretion induced by treatment of hyperlipidemic patients with the bile acid sequestrant cholestyramine for 7 years showed only a 13 and 20% decrease in plasma total cholesterol and LDL-C levels [87].

An interesting hypothesis of anti-atherosclerotic activity by metformin was introduced [88]. It was found that metformin increased expression of the fibroblast growth factor (FGF21) in hepatocytes, likely by the activation of AMPK [89], thereby stimulating expression of adenosine triphosphate binding cassette (ABC) transporters A1 and G1. This may increase cholesterol efflux from macrophages and decrease development of atherosclerotic plaques [88]. FGF21 is an important metabolic regulator, which may serve as a protection response against glucose-lipid disorders. The effects of metformin on FGF21 need further investigation, since it was reported that plasma FGF21 levels in humans with T2DM [90] are decreased after metformin treatment (opposite to the description in the hypothesis).

Another alternative pathway via which metformin may influence lipid metabolism in T2DM patients was proposed in [91]. Metformin induced activation of AMPK in the liver inhibited the SREBP-1c. The SREBP-1c gene was also found to be downregulated by metformin in another study [79]. This downregulation activated fatty acid desaturase 1(FADS1) and FADS2, which reduced arachidonic acid levels [92]. This reduction may cause increased membrane fluidity, thereby increasing LDL-C-receptor recycling and a reduction in the LDL-C levels [92].

Downregulation of SREBP1c affects many lipogenic genes. The acetyl-CoA carboxylase (ACC), catalysing the malonyl-CoA biosynthesis, was inhibited by AMPK during metformin exposure (0.5, 1, 2 mM; 27 h) in human hepatoma HepG2 cells [93], leading to a reduced amount of triglycerides. The gene fatty acid synthase (FASN) and SREBP-1C were also downregulated [75]. This indicates that the lipogenesis pathway may also be affected by metformin resulting in decreased fatty acids and triglycerides.

Figure 4 Summary of the effects of metformin in the liver that cause an overall improved lipid metabolism by reducing triglycerides, LDL-C, and total cholesterol.

### (In)-direct effects of metformin on β cells

Clearly a decreased β cell mass is an important factor in the development of T2DM. Gluco- and lipotoxicity (high glucose and FFA) induce damaging effects on β cells (e.g. decreased insulin secretion and β cell mass) [94]. It is therefore of interest to consider possible beneficial effects of metformin on β cell function. Research in this field is growing.

The enzymes lipase and amylase are secreted by the pancreas and are often measured to monitor the condition of the pancreas. There were no changes observed in lipase, amylase, and the pancreas volume when metformin (1950 mg/day) was given to T2DM patients for 24 weeks [95] suggesting that metformin does not repair damaged β cells. In this study, the product of dynamic, static and total β cell responsiveness and insulin sensitivity, also called the disposition indices (DI_d_, DI_s_, and DI_totOB_) calculated by an oral minimal model [96], showed that metformin (1000–1500 mg twice daily) for 2 weeks caused a significant increase in DI_d_, DI_s_, and DI_totOB_, a decreased homeostasis model assessment of insulin resistance (HOMA-IR), and an increased insulin sensitivity, majorly whole-body insulin sensitivity [97]. In contrast, another study showed no significant changes, which may perhaps occur because of the different personalized responses to metformin resulting in high standard errors [95]. The β cell responsivity was not altered in both studies and it was also suggested that metformin gives a more robust response to a high-fat mixed meal. This is also confirmed when treatment of metformin (1.45*10^−5^ M) showed to prevent damaging effects when human pancreatic islets were incubated with FFAs by restoring the insulin secretion dynamics [98]. Summarizing, metformin showed to increase the insulin sensitivity, but not β cell function.

Metformin was reported to exert beneficial effects in INS-1E cells (cell line which displays characteristics of the β cell). When these cells were exposed to 0.5 mM metformin for 24 h an increase in AMPK phosphorylation, a 30% reduction of SREBP-1C protein expression, a 80% increase of GLPR protein levels, and increased insulin promoter factor 1 (PDX-1) protein levels were observed [99]. The increase in AMPK phosphorylation may suppress c-jun N-terminal kinase (JNK) and p38 MAPK, resulting in reduced β cell apoptosis (2*10^−3^ M metformin INS-1E cells) [100]. However, in another study, metformin showed no effects on β cell survival nor β cell death in INS-1 cells, and it was found that GLP-1 (through a PKA and PI3K pathway) is able to reduce apoptosis [101]. As discussed previously, metformin treatment showed increased GLP-1 levels from the intestine, and this may explain the finding in the β cells.

Metformin treatment may also effect the compound nitric oxide (NO) and NO synthase (NOS) system, which play a significant role in β cell functioning and viability [102]. There is the neuronal constitutive NO synthase (ncNOS) which is associated with the mitochondria and insulin secretory granules, while inducible NOS (iNOS) located in the cytoplasm contributes to β cell failure during gluco- and lipotoxicity [102, 103]. Metformin (20*10^−3^ M) treatment had no effect on the NO-NOS system and insulin secretion in human and murine islets incubated at 7 mM glucose for 60 min [104]. However, metformin showed significant reduced ncNOS, iNOS, and total NOS activities, and slightly increased insulin secretion when the islets were incubated at 20 mM glucose for 60 min [104]. Metformin (0.5*10^−3^ M) prevents glycogen accumulation in INS-1 cells incubated with 25 mM glucose [105].

Summarizing, the available literature suggests that metformin ameliorates the damaging effects of high glucose and FFA in β cells, and that the NO-NOS system may play a role in regulating the insulin secretion. Studies to investigate the effects of metformin on β cells in more detail are ongoing [106].

## Potential mechanisms by which lipid lowering statins may affect glucose metabolism

### Introduction

Statins are a class of drugs that decrease plasma cholesterol levels and are prescribed as first choice to patients suffering from cardiovascular disease [107]. Simvastatin and atorvastatin are often given as a first choice to patients with cardiovascular risk factors/cardiovascular disease. Low dose (20 mg/day) atorvastatin therapy given to patients with myocardial infarction showed improved lipid, adipokine, and pro-inflammatory markers and decreased insulin resistance while higher dose (40 mg/day) of atorvastatin showed hyperglycemia, increased leptin levels and ghrelin deficiency [108, 109]. A meta-analysis observed that both intensive and non-intensive statin therapy may negatively influence glucose metabolism resulting in around 9% increased risk of developing new onset T2DM [110]. Recently, it was discovered that the reduction in LDL-C by statins is an important indicator of increased T2DM risk [111]. A LDL-C reduction between 30 and 40% induced a 13% increased risk of incident diabetes, the risk increasing to 29% with a 40–50% LDL-C reduction [111]. Genetic factors and/or age-related factors could as well lead to the development of T2DM during statin treatment.

In this review, the focus is to investigate the effects of statins on glucose metabolism. We focus on liver and pancreas because of their important role in glucose metabolism. However, statins may also act their worsening effect on glycemic control via other organs (intestine) and tissues (muscle and adipose tissue).

### Effects of statins on the endogenous glucose production in the liver

**Fig. 5:**
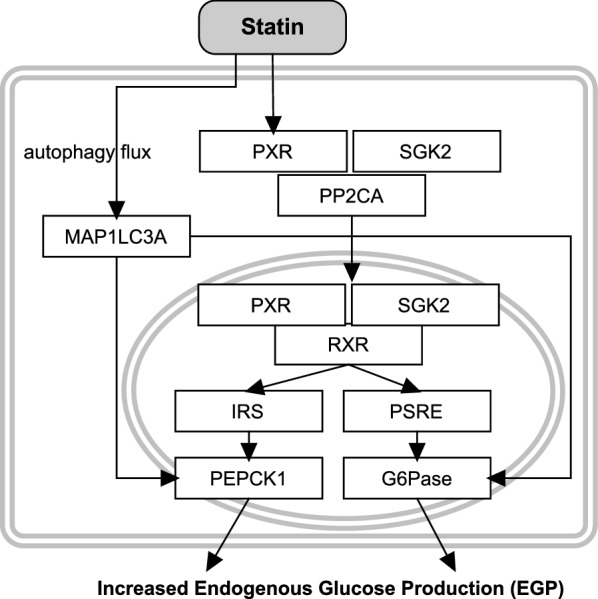
Schematic representation of the mechanisms of statins related to an increased EGP

Several mechanisms possibly involved in the effect of statins on glucose metabolism are summarized below and in Fig. 5.

A proposed statin signalling pathway that stimulates EGP by activation of gluconeogenic genes was discovered in human liver cells [112]. Statin activates the pregnane X receptor (PXR) in the cytoplasm. PXR exerts a number of functions, such as the stimulation of the expression of proteins involved in removal of xenobiotics, and regulation of hepatic glucose and lipid metabolism [113]. PXR binds to the serum/glucocorticoid regulated kinase 2 (SGK2) which stimulates dephosphorylation of protein phosphatase 2CA (PP2CA) [112]. As a result, PXR together with the dephosphorylated SGK2 move to the region where gluconeogenic genes are located in the nucleus. These regions are called the PXR–SGK2 response elements (PSRE) and an insulin response sequence region (IRS). PXR, SGK2 together with the nuclear retinoid X receptor (RXR) bind to these regions and thereby activate PEPCK1 and G6Pase [112]. This may result in an activation of EGP. Additionally, increased expression of PEPCK1 and G6Pase is observed through activation of an autophagic flux. Autophagy is a regulated destructive process of cellular elements and is upregulated for example during starvation, ER stress, or intracellular stress [114]. Contradictory results were found in a different study, where neither PEPCK1, G6Pase, nor EGP were affected in HepG2 cells treated with atorvastatin (1 and 10 μM) [115].

In vivo experiments investigating the effects of statin treatment on glucose metabolism in T2DM patients showed no remarkable effects on EGP. Basal EGP in T2DM patients treated with atorvastatin (10 mg; 12 weeks) [116] or simvastatin (80 mg/day; 8 weeks) [117] showed no changes. However, the EGP measured during clamp (isoglycaemic hyperinsulinaemic) conditions after 12 weeks of statin treatment was slightly increased compared with the baseline value in [116], but not in [117].

Summarizing, an increase of EGP induced by statins is not obvious from the above-mentioned studies performed in statin-treated T2DM patients, while it is observed in in vitro experiments. Therefore, it may be that the effects of statins on EGP are minor.

### Effects of statins in the β cell

**Fig. 6:**
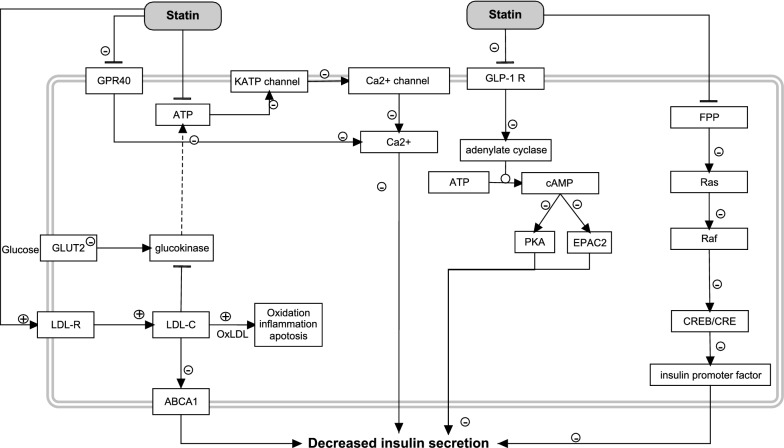
Hypothetical mechanisms related to a decreased insulin secretion in β cells induced by statins

In the literature, many statin-effected processes are described that may contribute to a decreased insulin secretion in the β cell, possibly contributing to the progress of T2DM (Fig. 6). One of these directly affected processes are the upregulation of LDL-C receptor seen upon inhibition of HMG-CoA reductase, which results in increased uptake of plasma LDL-C into the β cell [118]. The increased amount of cholesterol within the cell causes interference with translocation of glucokinase, to the mitochondria [119]. A decreased glucose transporter (GLUT2) expression level was observed in simvastatin treated mouse MIN6 cells which resulted in a reduction of ATP levels, inhibition of the K_ATP_ channel closure, membrane depolarization and calcium channel opening all leading to reduced insulin secretion [120]. The ATP-binding cassette transporter ABCA1 could also play an important role since a relation was discovered between ABCA1 deficiency and an impaired insulin secretion in the β cell [121]. Inhibition of the ATP-dependent potassium channel, depolarization and the decreased influx of calcium, and intracellular calcium concentrations were observed and were related to a decreased insulin secretion [122]. However, intracellular calcium levels were not affected in an ex vivo study wherein intact single-islets were treated with simvastatin [123].

Mouse MIN6 β cells treated with simvastatin (14.3 μM) showed inhibition of GPR40 (fatty acid receptor), and minor inhibition of GLP-1 resulting in a cascade of reactions leading to a decreased insulin secretion [122]. Atorvastatin treatment of rat INS-1 β cells induced inhibition of insulin synthesis by inhibiting farnesyl pyrophosphate ester (FPP, an intermediate in mevalonate and non-mevalonate pathways), which may inhibit a chain of proteins that communicates signals from the receptor to the nucleus and is called the Ras pathway (Ras/Raf/ERK/CREB) [124]. Inhibition of this pathway leads to inhibition of promoter activity of the insulin gene and to a decrease of insulin secretion [124]. Insulin secretion may also be impaired via direct statin induced inhibition of mitochondrial oxidative phosphorylation at complex III [125]. The resulting decrease in ATP synthesis may induce inhibition of insulin secretion via the cascade described above.

An interesting observation is that simvastatin treatment (40 mg/day; 6 weeks) in 148 patients resulted in a decreased β cell function (53%) and an increased insulin resistance (54%) [126]. One of the mechanisms of an increased insulin resistance could be the effect on the glucose transporter GLUT-4 located in adipose tissue and muscle. Atorvastatin treatment was shown to reduce the surface expression of GLUT-4 in mice adipocytes by inhibiting isoprenylation via inhibition of the mevalonate pathway [127]. Mevalonate is an important intermediate in cholesterol synthesis and hence also for the synthesis of isoprenoid intermediates (Ras and Rho proteins important in cell proliferation). These are involved in intracellular mobilization and localization of proteins. Statin treatment may cause inactivation of Ras and Rho molecules so that activation and membrane translocalization of GLUT-4 is inhibited. Experiments in mouse adipocytes confirmed that GLUT-4 located on the plasma membrane moved to the cytosol during atorvastatin treatment [128]. This may result in an increased insulin resistance.

In conclusion, statin treatment may lead to a decreased insulin secretion in the β cell via several mechanisms. However, these effects are up to now mainly seen in (animal) in vitro studies and so it remains elusive whether these results can be translated to humans. In addition, it should also be kept in mind that 10 years of statin treatment in patients caused an increased BMI (1.3 kg/m^2^ vs 0.4 kg/m^2^ in non-users) [129], and BMI is linked to insulin resistance which is an important factor in developing T2DM. It is not clear whether the patients that developed T2DM on statin treatment increased their BMI excessively. This is in contrast with metformin. Metformin treated mice showed a decreased weight gain which was related to the increased energy consuming conversion of glucose to lactate in the intestinal wall [130].

## Metformin–statin combination therapy

In diabetic rats (200–220 g) it was shown that after 2 weeks metformin–atorvastatin combination therapy (500 mg metformin and 20 mg atorvastatin per 70 kg body weight), glucose-lowering effects, lipid-lowering effects, reduction of oxidative stress, and positive effects on cardiovascular hypertrophy occurred [131]. The reduction of oxidative stress and protection of the liver (observed by studying the liver histology and blood measurement, e.g. CRP, TNF-α, IL-6, protein carbonyl levels) was also seen in T2DM rats treated with metformin and atorvastatin [132, 133]. These positive effects and the fact that a great number of patients are treated with Metformin**–**statin combination therapy led to the design of a metformin–atorvastatin combination tablet used as a single daily dose [134, 135]. There is only a minor chance for toxic drug interactions when metformin and statin are administered together because metformin is not metabolised and most statins are metabolised via the cytochrome P450 system [136].

Patients with T2DM are often taking metformin and statins together to control CVD risk as well as glucose metabolism [82]. Since metformin shows beneficial effects on both dyslipidemia and glycemic control and has been shown to reduce CVD risk while statins may have an added beneficial effect on CVD risk, combined treatment with both drugs seems a good option. As far as we have been able to discern no randomised clinical trials have been carried out to establish whether combination therapy is superior to monotherapy when focusing on CVD risk. Ethical considerations maybe prohibitive in this respect but perhaps subgroup analysis in ongoing studies such as the DDPOS may provide an answer. Studies aiming at optimal dosing of both drugs have not been performed. Clinical studies on the effects of metformin and statin combination therapy have been carried out but for different purposes [82, 137–143]. Each of these studies had different objectives and included different patients groups, i.e. either with T2DM, dyslipidemia, treated (different doses), untreated, or newly diagnosed T2DM. This precludes comparing these studies to arrive at overall results of metformin statin combination therapy.

Table 5 shows the glucose and lipid parameters after different doses combination therapy in mixed groups of patients (impaired fasting glucose (IFG)/dyslipidemia/T2DM). These studies are now discussed briefly to obtain knowledge about the overall effects on glucose and lipid metabolism in T2DM patients with dyslipidemia (Table 5). Surprisingly, the lowest dose of metformin (500 mg) and atorvastatin (10 mg) once daily resulted in the highest reduction of fasting plasma glucose (− 35%). Atorvastatin 20 mg showed to attenuate the glucose- and HbA1c-lowering effect in combination with 1000 and 2000 mg metformin. A high daily dose of simvastatin (40 mg) and metformin (3000 mg) resulted in an improved insulin resistance, but fasting plasma glucose decreased only by 5%, and minor changes were observed on lipid metabolism parameters, but this was probably due to the fact that metformin was given on top of simvastatin treatment. The patients in these studies had different diseased states, such as an impaired fasting glucose, dyslipidemia, newly diagnosed T2DM and/or dyslipidemia. This may complicate analysis of the obtained changes on the glucose and lipid metabolism. However, it could be used for hypothesis-generation rather than making rigid decisions, considering the lack of multiple dose dependent combination studies.

**Table 5:** Observed percentage changes of glucose and lipid variables of patients during combination therapy of metformin (M) and atorvastatin (A)/simvastatin (S)

Nr.	Study ref.	Treatment before the study	Combination treatment	Daily doses	Time (months)	N	Differences: end of the study versus baseline (%)									Subject type
							BMI	HOMA IR	FFA	FPG	HbA1c	TG	TC	LDL	HDL	
1	[138]	40 mg S	S + P	40 mg S	3	21		− 2	− 2	1 (6.5)		− 1 (1.39)				Patients with isolated IFG^a^, who because of hypercholesterolemia and/or coronary artery disease were treated for at least 3 months with S (40 mg daily), thus have a normal lipid profile (TC < 5.17 mmol/l, LDL < 3.36 mmol/l, TG < 1.69 mmol/l)
		40 mg S	S + M	40 mg S + 3 × 1000 mg M	3	20		− 62	− 25	− 5 (6.6)		− 12 (1.37)				
2	[139]	40 mg S	S + P	40 mg S	3	29		10	− 2	3 (6.2)	2					
		40 mg S	S + M	40 mg S + 3 × 1000 mg M	3			− 57	− 32	− 5 (6.3)	− 8					
3	[140]	40 mg S	S + P	40 mg S	3	29		4	− 3	2 (6.3)	0	2 (1.45)	1	1	− 2	
		40 mg S	S + M	40 mg S + 3x 1000 mg M	3	24		− 55	− 24	− 5 (6.4)	− 11	− 14 (1.41)	− 2	− 3	8	
4	[141]	–	M	850 mg M	3	17		− 22		− 11	− 17	− 1	10		6	Newly diagnosed T2DM with/without hypercholesterolemia (9/17 patients) and/or family history of DM (9/17)
		–	M + A	850 mg M + 10 mg A	3	18		− 2		− 15	− 19	− 23	− 28		− 10	Newly diagnosed T2DM with/without hypercholesterolemia (14/17 patients) and/or family history of DM (15/18)
5	[142]	–	M	850 mg M	1.5	17				~− 10 (9.3)	− 3	− 22 (1.35)		− 28	− 6.5	Newly diagnosed T2DM with/without hypercholesterolemia (9/17 patients) and/or family history of DM (9/17)
		–	M + A	850 mg M + 10 mg A	1.5	15				~− 13 (9.7)	− 16	− 15 (1.7)		− 36	− 11	Newly diagnosed T2DM with/without hypercholesterolemia (14/15 patients) and/or family history of DM (15/15)
6	[137]	Metformin/statin/−	M + A	500 mg M + 10 mg A	3	213	− 7			− 35 (10.2)	− 23.1	− 24	− 31	− 35	9	T2DM patients receiving metformin slow release and newly diagnosed with dyslipidemia or on atorvastatin with newly diagnosed diabetes or patients with newly diagnosed diabetic dyslipidemia. BMI ≥ 25, FPG levels between 7.8 and 13.9 mmol/l, postprandial glucose levels > 11.1 mmol/l, HbA1c levels between 7–12%
7	[82]	Metformin	M + A	2*1000 mg M + 20 mg A	3	109	− 2			− 8 (7.8)	− 1	− 12 (2.1)	− 9	− 17	3	Patients with a diagnosis of T2DM and treated with metformin
8	[143]	–	A	20 mg A	2	65	− 7			− 3 (5.9)	2	− 10 (1.9)	− 7	− 16	0	Non-diabetic overweight/obese patients with dyslipidemia
		–	M + A	2*500 mg M + 20 mg A	2	65	− 6			− 2 (5.7)	2	− 33 (1.9)	− 10	− 16	10	Non-diabetic overweight/obese patients with dyslipidemia

*P* placebo, *HOMA IR* homeostatic model assessment insulin resistance, *FFA* free fatty acids, *FPG* fasting plasma glucose, *TG* triglycerides, *TC* total cholesterol, *LDL* low-density lipoprotein, *HDL* high-density lipoprotein. Values between brackets for FPG and TG indicate baseline values in mmol/l

^a^Isolated impaired fasting glucose (IFG) indicates a FPG between 5.6 and 7 mmol/l and a plasma glucose 2 h after a 75 g OGTT below 7.8 mmol/l

The effects of metformin on lipid homeostasis as discussed in this review article, indicate that lipid metabolism is positively affected in the intestine and liver leading to decreased plasma triglycerides, LDL-C, and total cholesterol. Metformins effects on lipid metabolism seem to be localized to the intestine. Statins mainly act on plasma cholesterol levels via activation of the LDL-receptor suggesting that combination therapy should show an additional effect on plasma lipids. However, the data in Table 5 give little indication for an added beneficial effect of both drugs on lipid parameters. Dedicated studies are required to further investigate the effects of both drugs by combination therapy in humans. Metformin has been shown to exert a significant influence on the composition of the gut microbiota. Interestingly, statins showed such effects as well, particularly in studies with mice and rats [144, 145]. Statins were able to decrease the production of butyrate which may relate to the development of new onset T2DM [144]. Atorvastatin given to hypercholesterolemic patients restored anti-inflammatory bacteria [146].

Combination therapy with statins and metformin demonstrated beneficial effects in patients with other disease(s)/disorder(s) than T2DM and dyslipidemia. In T2DM patients with non-alcoholic fatty liver disease (NAFLD) beneficial use of combination therapy seems indicated since statin therapy associates negatively with non-alcoholic steatohepatitis and significant fibrosis while a safe use of metformin in patients with T2DM and NAFLD was demonstrated [147]. Combination therapy consisting of metformin and statin treatment is frequently prescribed to women with an endocrine disorder called polycystic ovary syndrome (PCOS). PCOS increases the risk of T2DM and cardiovascular morbidity as it is associated with abnormal increased lipid levels, insulin resistance, systemic inflammation and endothelial dysfunction [148]. Meta-analysis showed that combined statin-metformin therapy in women with PCOS resulted in improved lipid and inflammation markers but it did not improve insulin sensitivity [149]. Additional studies are recommended to confirm these results. Combination therapy could also be considered for T2DM patients with diabetic retinopathy. Diabetic retinopathy (DR) is a microvascular complication of diabetes caused by hyperglycemia and hyperosmolarity. Leakage and accumulation of fluid in the macula is known as macular edema and results in severe vision loss in DR patients. The use of statins in T2DM patients and pre-existing DR showed a protective effect against development of diabetic macular edema [150].

Remarkable is that T2DM patients receiving statin therapy in combination with increased levels of cholesterol remnants and triglycerides were associated with slight decreased in left ventricular systolic function. Targeting cholesterol remnants in addition to T2DM patients receiving statins might be beneficial on cardiac function [151]. From a clinical perspective, it was shown that many patients with T2DM and CVD did not receive lipid lowering therapy while their lipid levels were not in the optimal range [152]. Increased implementation of guideline recommendations for dyslipidemic T2DM patients is therefore recommended [152].

## Conclusion

Metformin is generally thought to exert its beneficial effects on glucose metabolism mainly in the liver. In line with recent literature on the topic we conclude that the drug acts primarily in the intestine. This is due to the at least one order of magnitude higher concentrations of metformin in the intestine than in the liver. The drug is certainly not absent in the liver hence parts of its effects may be localized to this organ most probably via its effects on gluconeogenesis. To treat T2DM and its cardiovascular comorbidity combination therapy of metformins with statins seems well placed and may act as a double-sided sword particularly in the case of statins. This drug increases the risk on T2DM particularly in prediabetic subjects, and cotreatment with metformin might reduce this risk. However, this hypothesis has not yet been systematically verified. In this review, we have investigated possible sites of interaction of metformin and statins and conclude that they act on largely parallel pathways. Statins reduce plasma cholesterol via activation of LDL-C receptor in the liver and may influence glucose homeostasis primarily by inhibition of insulin secretion in pancreatic β cells. We propose that combination therapy will ameliorate the risk of statin induced T2DM. More studies of combined Metformin**–**statin treatment in patients are necessary to explain the effects of different Metformin**–**statin doses on the glucose and lipid metabolism, in different stages of progression of diabetes and/or dyslipidemia, in more detail.

## Abbreviations

ABCA1: ATP-binding cassette transporter/cholesterol efflux regulatory protein (CERP)
ACAT: Acyl-CoA cholesterol acyltransferase
ACC1: acetyl-CoA carboxylase
ASBT: apical sodium-dependent bile acid transporter
BA: bile acids
BMI: body mass index
CAMK2: calcium–calmodulin dependent kinase 2
CREB: cAMP response element binding protein
CVD: cardiovascular disease
DI: disposition indice
DR: diabetic retinopathy
EGP: endogenous glucose production
FADS: fatty acid desaturase
FAS: fatty acid synthase
FFA: free fatty acid
FGF: fibroblast growth factor
FPP: farnesyl pyrophosphate
FXR: farnesoid X receptor
G6Pase: glucose-6-phosphatase
GLP-1: glucagon-like peptide-1
GLPR: GLP-1 receptor
GLUT: glucose transporter
GSK-3b: glycogen synthase kinase 3
HbA1c: glycated hemoglobin
HDL-C: high-density lipoprotein cholesterol
HMGCR: 3-hydroxy-3-methyl-glutaryl-coenzyme A reductase
HMGCS: HMG-CoA synthase
HOMA-IR: homeostasis model assessment of insulin resistance
IFG: impaired fasting glucose
iNOS: inducible NOS
IRS2: insulin receptor substrate 2
JNK: c-jun N-terminal kinase
LDL-C: low-density lipoprotein
mGPD: mitochondrial glycerol-3-phosphate dehydrogenase
NMDA: *N*-methyl-d-aspartate
ncNOS: neuronal constitutive NO synthase
NO: nitric oxide
NOS: NO synthase
OCT: organic cation transporter
PDX: insulin promoter factor
PEPCK: phosphoenolpyruvate carboxykinase
PI3K: phosphoinositide 3 kinase
PKA: protein kinase A
PKB/AKT: protein kinase B
PMAT: plasma monoamine transporter
PP2CA: protein phosphatase 2CA
PSRE: PXR–SGK2 response elements
PXR: pregnane X receptor
RXR: retinoid X receptor
SERT: sodium-dependent serotonin transporter
SGK: serum/glucocorticoid regulated kinase
SGLT: sodium/glucose cotransporter
SREBP: sterol regulatory element-binding protein
T2DM: type 2 diabetes mellitus
TCF4L2: transcription factor 7-like 2
TG: triglycerides
TGR5: G protein-coupled bile acid receptor 1
THTR: thiamine transporter

## References

[1] Bosi E (2009). Metformin—the gold standard in type 2 diabetes: what does the evidence tell us?. Diabetes Obes Metab.

[2] Wu L, Parhofer KG (2014). Diabetic dyslipidemia. Metabolism.

[3] Grundy SM, Brewer HB, Cleeman JI, Smith SC, Lenfant C, American Heart A, National Heart L, Blood I (2004). Definition of metabolic syndrome: report of the National Heart, Lung, and Blood Institute/American Heart Association conference on scientific issues related to definition. Circulation.

[4] Kitao N, Miyoshi H, Furumoto T, Ono K, Nomoto H, Miya A, Yamamoto C, Inoue A, Tsuchida K, Manda N (2017). The effects of vildagliptin compared with metformin on vascular endothelial function and metabolic parameters: a randomized, controlled trial (Sapporo Athero-Incretin Study 3). Cardiovasc Diabetol.

[5] Mortensen MB, Kulenovic I, Falk E (2016). Statin use and cardiovascular risk factors in diabetic patients developing a first myocardial infarction. Cardiovasc Diabetol.

[6] Sattar N, Preiss D, Murray HM, Welsh P, Buckley BM, de Craen AJ, Seshasai SR, McMurray JJ, Freeman DJ, Jukema JW (2010). Statins and risk of incident diabetes: a collaborative meta-analysis of randomised statin trials. Lancet.

[7] Corrao G, Ibrahim B, Nicotra F, Soranna D, Merlino L, Catapano AL, Tragni E, Casula M, Grassi G, Mancia G (2014). Statins and the risk of diabetes: evidence from a large population-based cohort study. Diabetes Care.

[8] Fischer J, Ganellin CR, Ganesan A, Proudfoot J, Ganellin JFACR (2010). Standalone drugs. Analogue-based drug discovery.

[9] Timmins P, Donahue S, Meeker J, Marathe P (2005). Steady-state pharmacokinetics of a novel extended-release metformin formulation. Clin Pharmacokinet.

[10] Buse JB, DeFronzo RA, Rosenstock J, Kim T, Burns C, Skare S, Baron A, Fineman M (2016). The primary glucose-lowering effect of metformin resides in the gut, not the circulation: results from short-term pharmacokinetic and 12-week dose-ranging studies. Diabetes Care.

[11] Hashimoto Y, Tanaka M, Okada H, Mistuhashi K, Kimura T, Kitagawa N, Fukuda T, Majima S, Fukuda Y, Tanaka Y (2016). Postprandial hyperglycemia was ameliorated by taking metformin 30 min before a meal than taking metformin with a meal; a randomized, open-label, crossover pilot study. Endocrine.

[12] Hernandez B, Pfluger F, Kruglik SG, Cohen R, Ghomi M (2015). Protonation-deprotonation and structural dynamics of antidiabetic drug metformin. J Pharm Biomed Anal.

[13] Bretnall AE, Clarke GS (1998). Metformin hydrochloride. Anal Profiles Drug subst Excipients.

[14] Orgovan G, Noszal B (2011). Electrodeless, accurate pH determination in highly basic media using a new set of (1)H NMR pH indicators. J Pharm Biomed Anal.

[15] Wilcock C, Wyre ND, Bailey CJ (1991). Subcellular distribution of metformin in rat liver. J Pharm Pharmacol.

[16] Wiernsperger NF (1999). Membrane physiology as a basis for the cellular effects of metformin in insulin resistance and diabetes. Diabetes Metab.

[17] Kinaan M, Ding H, Triggle CR (2015). Metformin: an old drug for the treatment of diabetes but a new drug for the protection of the endothelium. Med Princ Pract.

[18] Bridges HR, Sirvio VA, Agip AN, Hirst J (2016). Molecular features of biguanides required for targeting of mitochondrial respiratory complex I and activation of AMP-kinase. BMC Biol.

[19] Chien HC, Zur AA, Maurer TS, Yee SW, Tolsma J, Jasper P, Scott DO, Giacomini KM (2016). Rapid method to determine intracellular drug concentrations in cellular uptake assays: application to metformin in organic cation transporter 1-transfected human embryonic kidney 293 cells. Drug Metab Dispos.

[20] He L, Wondisford FE (2015). Metformin action: concentrations matter. Cell Metab.

[21] Wilcock C, Bailey CJ (1990). Sites of metformin-stimulated glucose metabolism. Biochem Pharmacol.

[22] Gormsen LC, Sundelin EI, Jensen JB, Vendelbo MH, Jakobsen S, Munk OL, Christensen MM, Brosen K, Frokiaer J, Jessen N (2016). In vivo imaging of human 11C-metformin in peripheral organs: dosimetry, biodistribution and kinetic analyses. J Nucl Med.

[23] Han TK, Proctor WR, Costales CL, Cai H, Everett RS, Thakker DR (2015). Four cation-selective transporters contribute to apical uptake and accumulation of metformin in Caco-2 cell monolayers. J Pharmacol Exp Ther.

[24] McCreight LJ, Bailey CJ, Pearson ER (2016). Metformin and the gastrointestinal tract. Diabetologia.

[25] Liang X, Chien HC, Yee SW, Giacomini MM, Chen EC, Piao M, Hao J, Twelves J, Lepist EI, Ray AS (2015). Metformin is a substrate and inhibitor of the human thiamine transporter, THTR-2 (SLC19A3). Mol Pharm.

[26] Zhou M, Xia L, Wang J (2007). Metformin transport by a newly cloned proton-stimulated organic cation transporter (plasma membrane monoamine transporter) expressed in human intestine. Drug Metab Dispos.

[27] Dujic T, Zhou K, Donnelly LA, Tavendale R, Palmer CN, Pearson ER (2015). Association of organic cation transporter 1 with intolerance to metformin in type 2 diabetes: a GoDARTS study. Diabetes.

[28] Muller J, Lips KS, Metzner L, Neubert RH, Koepsell H, Brandsch M (2005). Drug specificity and intestinal membrane localization of human organic cation transporters (OCT). Biochem Pharmacol.

[29] Proctor WR, Ming X, Bourdet D, Han TK, Everett RS, Thakker DR (2016). Why does the intestine lack basolateral efflux transporters for cationic compounds? A provocative hypothesis. J Pharm Sci.

[30] Bailey CJ, Wilcock C, Scarpello JH (2008). Metformin and the intestine. Diabetologia.

[31] Duez H, Lamarche B, Uffelman KD, Valero R, Cohn JS, Lewis GF (2006). Hyperinsulinemia is associated with increased production rate of intestinal apolipoprotein B-48-containing lipoproteins in humans. Arterioscler Thromb Vasc Biol.

[32] Xiao C, Dash S, Morgantini C, Adeli K, Lewis GF (2015). Gut peptides are novel regulators of intestinal lipoprotein secretion: experimental and pharmacological manipulation of lipoprotein metabolism. Diabetes.

[33] Gutierrez-Repiso C, Rodriguez-Pacheco F, Garcia-Arnes J, Valdes S, Gonzalo M, Soriguer F, Moreno-Ruiz FJ, Rodriguez-Cañete A, Gallego-Perales JL, Alcain-Martinez G (2015). The expression of genes involved in jejunal lipogenesis and lipoprotein synthesis is altered in morbidly obese subjects with insulin resistance. Lab Invest.

[34] Jeppesen J, Zhou MY, Chen YD, Reaven GM (1994). Effect of metformin on postprandial lipemia in patients with fairly to poorly controlled NIDDM. Diabetes Care.

[35] Field FJ, Born E, Murthy S, Mathur SN (2001). Gene expression of sterol regulatory element-binding proteins in hamster small intestine. J Lipid Res.

[36] Kohjima M, Higuchi N, Kato M, Kotoh K, Yoshimoto T, Fujino T, Yada M, Yada R, Harada N, Enjoji M (2008). SREBP-1c, regulated by the insulin and AMPK signaling pathways, plays a role in nonalcoholic fatty liver disease. Int J Mol Med.

[37] Srivastava RA, Pinkosky SL, Filippov S, Hanselman JC, Cramer CT, Newton RS (2012). AMP-activated protein kinase: an emerging drug target to regulate imbalances in lipid and carbohydrate metabolism to treat cardio-metabolic diseases. J Lipid Res.

[38] Tso P, Sun W, Liu M (2004). Gastrointestinal satiety signals IV. Apolipoprotein A-IV. Am J Physiol Gastrointest Liver Physiol.

[39] Lutz TA, Osto E (2016). Glucagon-like peptide-1, glucagon-like peptide-2, and lipid metabolism. Curr Opin Lipidol.

[40] Dash S, Xiao C, Morgantini C, Lewis GF (2015). New insights into the regulation of chylomicron production. Annu Rev Nutr.

[41] Harmel E, Grenier E, Bendjoudi Ouadda A, El Chebly M, Ziv E, Beaulieu JF, Sane A, Spahis S, Laville M, Levy E (2014). AMPK in the small intestine in normal and pathophysiological conditions. Endocrinology.

[42] Rajas F, Bruni N, Montano S, Zitoun C, Mithieux G (1999). The glucose-6 phosphatase gene is expressed in human and rat small intestine: regulation of expression in fasted and diabetic rats. Gastroenterology.

[43] Mithieux G, Gautier-Stein A (2014). Intestinal glucose metabolism revisited. Diabetes Res Clin Pract.

[44] Soty M, Penhoat A, Amigo-Correig M, Vinera J, Sardella A, Vullin-Bouilloux F, Zitoun C, Houberdon I, Mithieux G (2015). A gut-brain neural circuit controlled by intestinal gluconeogenesis is crucial in metabolic health. Mol Metab.

[45] Mithieux G, Rajas F, Zitoun C (2006). Glucose utilization is suppressed in the gut of insulin-resistant high fat-fed rats and is restored by metformin. Biochem Pharmacol.

[46] Forslund K, Hildebrand F, Nielsen T, Falony G, Le Chatelier E, Sunagawa S, Prifti E, Vieira-Silva S, Gudmundsdottir V, Krogh Pedersen H (2015). Disentangling type 2 diabetes and metformin treatment signatures in the human gut microbiota. Nature.

[47] Wu H, Esteve E, Tremaroli V, Khan MT, Caesar R, Manneras-Holm L, Stahlman M, Olsson LM, Serino M, Planas-Felix M (2017). Metformin alters the gut microbiome of individuals with treatment-naive type 2 diabetes, contributing to the therapeutic effects of the drug. Nat Med.

[48] Duca FA, Cote CD, Rasmussen BA, Zadeh-Tahmasebi M, Rutter GA, Filippi BM, Lam TK (2015). Metformin activates a duodenal Ampk-dependent pathway to lower hepatic glucose production in rats. Nat Med.

[49] Hardie DG (2004). AMP-activated protein kinase: a master switch in glucose and lipid metabolism. Rev Endocr Metab Disord.

[50] Coughlan KA, Valentine RJ, Ruderman NB, Saha AK (2014). AMPK activation: a therapeutic target for type 2 diabetes?. Diabetes Metab Syndr Obes.

[51] Ruderman NB, Carling D, Prentki M, Cacicedo JM (2013). AMPK, insulin resistance, and the metabolic syndrome. J Clin Invest.

[52] Sandoval DA, D’Alessio DA (2015). Physiology of proglucagon peptides: role of glucagon and GLP-1 in health and disease. Physiol Rev.

[53] Habener JF, Kieffer TJ, Ronald Kahn C (2005). Glucagon and glucagon-like peptides, chap. 11. Joslin’s diabetes mellitus.

[54] Mannucci E, Tesi F, Bardini G, Ognibene A, Petracca MG, Ciani S, Pezzatini A, Brogi M, Dicembrini I, Cremasco F (2004). Effects of metformin on glucagon-like peptide-1 levels in obese patients with and without type 2 diabetes. Diabetes Nutr Metab.

[55] Napolitano A, Miller S, Nicholls AW, Baker D, Van Horn S, Thomas E, Rajpal D, Spivak A, Brown JR, Nunez DJ (2014). Novel gut-based pharmacology of metformin in patients with type 2 diabetes mellitus. PLoS ONE.

[56] Preiss D, Dawed A, Welsh P, Heggie A, Jones AG, Dekker J, Koivula R, Hansen TH, Stewart MC, Consortium D (2016). The sustained influence of metformin therapy on circulating GLP-1 levels in individuals with and without type 2 diabetes. Diabetes Obes Metab.

[57] Rohde U, Sonne DP, Christensen M, Hansen M, Bronden A, Torang S, Rehfeld JF, Holst JJ, Vilsboll T, Knop FK (2016). Cholecystokinin-induced gallbladder emptying and metformin elicit additive glucagon-like peptide-1 responses. J Clin Endocrinol Metab.

[58] Kim MH, Jee JH, Park S, Lee MS, Kim KW, Lee MK (2014). Metformin enhances glucagon-like peptide 1 via cooperation between insulin and Wnt signaling. J Endocrinol.

[59] Liu YX, Si MM, Lu W, Zhang LX, Zhou CX, Deng SL, Wu HS (2015). Effects and molecular mechanisms of the antidiabetic fraction of *Acorus calamus* L. on GLP-1 expression and secretion in vivo and in vitro. J Ethnopharmacol.

[60] Trabelsi MS, Daoudi M, Prawitt J, Ducastel S, Touche V, Sayin SI, Perino A, Brighton CA, Sebti Y, Kluza J (2015). Farnesoid X receptor inhibits glucagon-like peptide-1 production by enteroendocrine L cells. Nat Commun.

[61] Jensen JB, Sundelin EI, Jakobsen S, Gormsen LC, Munk OL, Frokiaer J, Jessen N (2016). [11C]-Labeled metformin distribution in the liver and small intestine using dynamic positron emission tomography in mice demonstrates tissue-specific transporter dependency. Diabetes.

[62] Zamek-Gliszczynski MJ, Bao JQ, Day JS, Higgins JW (2013). Metformin sinusoidal efflux from the liver is consistent with negligible biliary excretion and absence of enterohepatic cycling. Drug Metab Dispos.

[63] Scarpello JH, Hodgson E, Howlett HC (1998). Effect of metformin on bile salt circulation and intestinal motility in type 2 diabetes mellitus. Diabet Med.

[64] Ryan P, Delzenne N, Hyland N, Stanton C (2016). Gut microbiota and metabolism. The Gut-Brain axis.

[65] Wang Z, Koonen D, Hofker M, Fu J (2016). Gut microbiome and lipid metabolism: from associations to mechanisms. Curr Opin Lipidol.

[66] Wahlstrom A, Sayin SI, Marschall HU, Backhed F (2016). Intestinal crosstalk between bile acids and microbiota and its impact on host metabolism. Cell Metab.

[67] de la Cuesta-Zuluaga J, Mueller NT, Corrales-Agudelo V, Velásquez-Mejía EP, Carmona JA, Abad JM, Escobar JS (2016). Metformin is associated with higher relative abundance of mucin-degrading *Akkermansia muciniphila* and several short-chain fatty acid-producing microbiota in the gut. Diabetes Care.

[68] Dao MC, Everard A, Aron-Wisnewsky J, Sokolovska N, Prifti E, Verger EO, Kayser BD, Levenez F, Chilloux J, Hoyles L (2016). Akkermansia muciniphila and improved metabolic health during a dietary intervention in obesity: relationship with gut microbiome richness and ecology. Gut.

[69] Zhou K, Pedersen HK, Dawed AY, Pearson ER (2016). Pharmacogenomics in diabetes mellitus: insights into drug action and drug discovery. Nat Rev Endocrinol.

[70] Wang DS, Jonker JW, Kato Y, Kusuhara H, Schinkel AH, Sugiyama Y (2002). Involvement of organic cation transporter 1 in hepatic and intestinal distribution of metformin. J Pharmacol Exp Ther.

[71] Foretz M, Guigas B, Bertrand L, Pollak M, Viollet B (2014). Metformin: from mechanisms of action to therapies. Cell Metab.

[72] Gruszka A (2016). New insight into the mechanisms of the anti-hyperglycemic action of metformin. Br J Med Med Res.

[73] Baur JA, Birnbaum MJ (2014). Control of gluconeogenesis by metformin: does redox trump energy charge?. Cell Metab.

[74] Viollet B, Guigas B, Leclerc J, Hebrard S, Lantier L, Mounier R, Andreelli F, Foretz M (2009). AMP-activated protein kinase in the regulation of hepatic energy metabolism: from physiology to therapeutic perspectives. Acta Physiol (Oxf).

[75] Madsen A, Bozickovic O, Bjune JI, Mellgren G, Sagen JV (2015). Metformin inhibits hepatocellular glucose, lipid and cholesterol biosynthetic pathways by transcriptionally suppressing steroid receptor coactivator 2 (SRC-2). Sci Rep.

[76] Zhou G, Myers R, Li Y, Chen Y, Shen X, Fenyk-Melody J, Wu M, Ventre J, Doebber T, Fujii N (2001). Role of AMP-activated protein kinase in mechanism of metformin action. J Clin Invest.

[77] Maziere JC, Maziere C, Mora L, Gardette J, Salmon S, Auclair M, Polonovski J (1988). The antidiabetic drug metformin decreases cholesterol metabolism in cultured human fibroblasts. Atherosclerosis.

[78] Koren-Gluzer M, Aviram M, Hayek T (2013). Metformin inhibits macrophage cholesterol biosynthesis rate: possible role for metformin-induced oxidative stress. Biochem Biophys Res Commun.

[79] Liu ZQ, Song XM, Chen QT, Liu T, Teng JT, Zhou K, Luo DQ (2016). Effect of metformin on global gene expression in liver of KKAy mice. Pharmacol Rep.

[80] Scott LM, Tomkin GH (1983). Changes in hepatic and intestinal cholesterol regulatory enzymes. The influence of metformin. Biochem Pharmacol.

[81] Chakraborty A, Chowdhury S, Bhattacharyya M (2011). Effect of metformin on oxidative stress, nitrosative stress and inflammatory biomarkers in type 2 diabetes patients. Diabetes Res Clin Pract.

[82] Kashi Z, Mahrooz A, Kianmehr A, Alizadeh A (2016). The role of metformin response in lipid metabolism in patients with recent-onset type 2 diabetes: HbA1c level as a criterion for designating patients as responders or nonresponders to metformin. PLoS ONE.

[83] Ohira M, Miyashita Y, Ebisuno M, Saiki A, Endo K, Koide N, Oyama T, Murano T, Watanabe H, Shirai K (2007). Effect of metformin on serum lipoprotein lipase mass levels and LDL particle size in type 2 diabetes mellitus patients. Diabetes Res Clin Pract.

[84] Zhang C, Gao F, Luo H, Zhang CT, Zhang R (2015). Differential response in levels of high-density lipoprotein cholesterol to one-year metformin treatment in prediabetic patients by race/ethnicity. Cardiovasc Diabetol.

[85] Sonne DP, Knop FK. Comment on Xu et al. Effects of metformin on metabolite profiles and LDL cholesterol in patients with type 2 diabetes. Diabetes Care 2015;38:1858–1867. Diabetes Care. 2015; 38(12):e215. 10.2337/dc15-1794 26604291

[86] Hofmann AF, Hagey LR (2014). Key discoveries in bile acid chemistry and biology and their clinical applications: history of the last eight decades. J Lipid Res.

[87] The lipid research clinics coronary primary prevention trial results. I. Reduction in incidence of coronary heart disease. JAMA. 1984;251(3):351–64. 10.1001/jama.1984.03340270029025 6361299

[88] Luo F, Guo Y, Ruan G, Li X (2016). Metformin promotes cholesterol efflux in macrophages by up-regulating FGF21 expression: a novel anti-atherosclerotic mechanism. Lipids Health Dis.

[89] Nygaard EB, Vienberg SG, Orskov C, Hansen HS, Andersen B (2012). Metformin stimulates FGF21 expression in primary hepatocytes. Exp Diabetes Res.

[90] Fan H, Sun X, Zhang H, Liu J, Zhang P, Xu Y, Pan Q, Wang G (2016). Effect of metformin on fibroblast growth factor-21 levels in patients with newly diagnosed type 2 diabetes. Diabetes Technol Ther.

[91] Xu T, Brandmaier S, Messias AC, Herder C, Draisma HH, Demirkan A, Yu Z, Ried JS, Haller T, Heier M (2015). Effects of metformin on metabolite profiles and LDL cholesterol in patients with type 2 diabetes. Diabetes Care.

[92] Sone Y, Kido T, Ainuki T, Sonoda M, Ichi I, Kodama S, Sone H, Kondo K, Morita Y, Egawa S (2013). Genetic variants of the fatty acid desaturase gene cluster are associated with plasma LDL cholesterol levels in Japanese males. J Nutr Sci Vitaminol (Tokyo).

[93] Zang M, Zuccollo A, Hou X, Nagata D, Walsh K, Herscovitz H, Brecher P, Ruderman NB, Cohen RA (2004). AMP-activated protein kinase is required for the lipid-lowering effect of metformin in insulin-resistant human HepG2 cells. J Biol Chem.

[94] Wang J, Yang X, Zhang J (2016). Bridges between mitochondrial oxidative stress, ER stress and mTOR signaling in pancreatic β cells. Cell Signal.

[95] Tanaka K, Saisho Y, Manesso E, Tanaka M, Meguro S, Irie J, Sugiura H, Kawai T, Jinzaki M, Cobelli C (2015). Effects of liraglutide monotherapy on beta cell function and pancreatic enzymes compared with metformin in Japanese overweight/obese patients with type 2 diabetes mellitus: a subpopulation analysis of the KIND-LM randomized trial. Clin Drug Investig.

[96] Cobelli C, Dalla Man C, Toffolo G, Basu R, Vella A, Rizza R (2014). The oral minimal model method. Diabetes.

[97] Konopka AR, Esponda RR, Robinson MM, Johnson ML, Carter RE, Schiavon M, Cobelli C, Wondisford FE, Lanza IR, Nair KS (2016). Hyperglucagonemia mitigates the effect of metformin on glucose production in prediabetes. Cell Rep.

[98] Lupi R, Del Guerra S, Fierabracci V, Marselli L, Novelli M, Patane G, Boggi U, Mosca F, Piro S, Del Prato S (2002). Lipotoxicity in human pancreatic islets and the protective effect of metformin. Diabetes.

[99] Natalicchio A, Biondi G, Marrano N, Labarbuta R, Tortosa F, Spagnuolo R, D’Oria R, Carchia E, Leonardini A, Cignarelli A (2016). Long-Term exposure of pancreatic beta-cells to palmitate results in SREBP-1C-dependent decreases in GLP-1 receptor signaling via CREB and AKT and insulin secretory response. Endocrinology.

[100] Dai YL, Huang SL, Leng Y (2015). AICAR and metformin exert AMPK-dependent effects on INS-1E pancreatic beta-cell apoptosis via differential downstream mechanisms. Int J Biol Sci.

[101] Chang TJ, Tseng HC, Liu MW, Chang YC, Hsieh ML, Chuang LM (2016). Glucagon-like peptide-1 prevents methylglyoxal-induced apoptosis of beta cells through improving mitochondrial function and suppressing prolonged AMPK activation. Sci Rep.

[102] Muhammed SJ, Lundquist I, Salehi A (2012). Pancreatic beta-cell dysfunction, expression of iNOS and the effect of phosphodiesterase inhibitors in human pancreatic islets of type 2 diabetes. Diabetes Obes Metab.

[103] Mezghenna K, Pomies P, Chalancon A, Castex F, Leroy J, Niclauss N, Nadal B, Cambier L, Cazevieille C, Petit P (2011). Increased neuronal nitric oxide synthase dimerisation is involved in rat and human pancreatic beta cell hyperactivity in obesity. Diabetologia.

[104] Lundquist I, Mohammed Al-Amily I, Meidute Abaraviciene S, Salehi A (2016). Metformin ameliorates dysfunctional traits of glibenclamide- and glucose-induced insulin secretion by suppression of imposed overactivity of the islet nitric oxide synthase-NO system. PLoS ONE.

[105] Brereton MF, Rohm M, Shimomura K, Holland C, Tornovsky-Babeay S, Dadon D, Iberl M, Chibalina MV, Lee S, Glaser B (2016). Hyperglycaemia induces metabolic dysfunction and glycogen accumulation in pancreatic beta-cells. Nat Commun.

[106] Consortium R (2014). Restoring insulin secretion (RISE): design of studies of beta-cell preservation in prediabetes and early type 2 diabetes across the life span. Diabetes Care.

[107] Bibbins-Domingo K, Grossman DC, Curry SJ, Davidson KW, Epling JW, Garcia FA, Gillman MW, Kemper AR, Krist AH, Force USPST (2016). Statin use for the primary prevention of cardiovascular disease in adults: US preventive services task force recommendation statement. JAMA.

[108] Gruzdeva O, Uchasova E, Dyleva Y, Akbasheva O, Karetnikova V, Shilov A, Barbarash O (2017). Effect of different doses of statins on the development of type 2 diabetes mellitus in patients with myocardial infarction. Diabetes Metab Syndr Obes Targets Ther.

[109] Gruzdeva O, Uchasova E, Dyleva Y, Akbasheva O, Karetnikova V, Barbarash O (2016). Early effects of treatment low-dose atorvastatin on markers of insulin resistance and inflammation in patients with myocardial infarction. Front Pharmacol.

[110] Anyanwagu U, Idris I, Donnelly R (2016). Drug-induced diabetes mellitus: evidence for statins and other drugs affecting glucose metabolism. Clin Pharmacol Ther.

[111] Wang S, Cai R, Yuan Y, Varghese Z, Moorhead J, Ruan XZ (2017). Association between reductions in low-density lipoprotein cholesterol with statin therapy and the risk of new-onset diabetes: a meta-analysis. Sci Rep.

[112] Gotoh S, Negishi M (2015). Statin-activated nuclear receptor PXR promotes SGK2 dephosphorylation by scaffolding PP2C to induce hepatic gluconeogenesis. Sci Rep.

[113] Hakkola J, Rysa J, Hukkanen J (2016). Regulation of hepatic energy metabolism by the nuclear receptor PXR. Biochim Biophys Acta.

[114] He C, Klionsky DJ (2009). Regulation mechanisms and signaling pathways of autophagy. Annu Rev Genet.

[115] Ling Z, Shu N, Xu P, Wang F, Zhong Z, Sun B, Li F, Zhang M, Zhao K, Tang X (2016). Involvement of pregnane X receptor in the impaired glucose utilization induced by atorvastatin in hepatocytes. Biochem Pharmacol.

[116] Black RN, Ennis CN, Young IS, Hunter SJ, Atkinson AB, Bell PM (2014). The peroxisome proliferator-activated receptor alpha agonist fenofibrate has no effect on insulin sensitivity compared to atorvastatin in type 2 diabetes mellitus; a randomised, double-blind controlled trial. J Diabetes Complications.

[117] Szendroedi J, Anderwald C, Krssak M, Bayerle-Eder M, Esterbauer H, Pfeiler G, Brehm A, Nowotny P, Hofer A, Waldhausl W (2009). Effects of high-dose simvastatin therapy on glucose metabolism and ectopic lipid deposition in nonobese type 2 diabetic patients. Diabetes Care.

[118] Ruscica M, Macchi C, Morlotti B, Sirtori CR, Magni P (2014). Statin therapy and related risk of new-onset type 2 diabetes mellitus. Eur J Intern Med.

[119] Hao M, Head WS, Gunawardana SC, Hasty AH, Piston DW (2007). Direct effect of cholesterol on insulin secretion: a novel mechanism for pancreatic beta-cell dysfunction. Diabetes.

[120] Zhou J, Li W, Xie Q, Hou Y, Zhan S, Yang X, Xu X, Cai J, Huang Z (2014). Effects of simvastatin on glucose metabolism in mouse MIN6 cells. J Diabetes Res.

[121] Vergeer M, Brunham LR, Koetsveld J, Kruit JK, Verchere CB, Kastelein JJ, Hayden MR, Stroes ES (2010). Carriers of loss-of-function mutations in ABCA1 display pancreatic beta-cell dysfunction. Diabetes Care.

[122] Yaluri N, Modi S, Lopez Rodriguez M, Stancakova A, Kuusisto J, Kokkola T, Laakso M (2015). Simvastatin impairs insulin secretion by multiple mechanisms in MIN6 cells. PLoS ONE.

[123] Scattolini V, Luni C, Zambon A, Galvanin S, Gagliano O, Ciubotaru CD, Avogaro A, Mammano F, Elvassore N, Fadini GP (2016). Simvastatin rapidly and reversibly inhibits insulin secretion in intact single-islet cultures. Diabetes Ther.

[124] Sun H, Li Y, Sun B, Hou N, Yang J, Zheng M, Xu J, Wang J, Zhang Y, Zeng X (2016). Atorvastatin inhibits insulin synthesis by inhibiting the Ras/Raf/ERK/CREB pathway in INS-1 cells. Medicine (Baltimore).

[125] Schirris TJ, Renkema GH, Ritschel T, Voermans NC, Bilos A, van Engelen BG, Brandt U, Koopman WJ, Beyrath JD, Rodenburg RJ (2015). Statin-induced myopathy is associated with mitochondrial complex iii inhibition. Cell Metab.

[126] Elbadawi-Sidhu M, Baillie RA, Zhu H, Chen YDI, Goodarzi MO, Rotter JI, Krauss RM, Fiehn O, Kaddurah-Daouk R (2017). Pharmacometabolomic signature links simvastatin therapy and insulin resistance. Metabolomics.

[127] Takaguri A, Satoh K, Itagaki M, Tokumitsu Y, Ichihara K (2008). Effects of atorvastatin and pravastatin on signal transduction related to glucose uptake in 3T3L1 adipocytes. J Pharmacol Sci.

[128] Nakata M, Nagasaka S, Kusaka I, Matsuoka H, Ishibashi S, Yada T (2006). Effects of statins on the adipocyte maturation and expression of glucose transporter 4 (SLC2A4): implications in glycaemic control. Diabetologia.

[129] Sugiyama T, Tsugawa Y, Tseng CH, Kobayashi Y, Shapiro MF (2014). Different time trends of caloric and fat intake between statin users and nonusers among US adults: gluttony in the time of statins?. JAMA Intern Med.

[130] Schommers P, Thurau A, Bultmann-Mellin I, Guschlbauer M, Klatt AR, Rozman J, Klingenspor M, de Angelis MH, Alber J, Gründemann D (2017). Metformin causes a futile intestinal—hepatic cycle which increases energy expenditure and slows down development of a type 2 diabetes-like state. Molecular Metabolism.

[131] Islam M, Alam A, Rahman M, Ali Y, Mamun A, Rahman M, Hossain A, Rashid M (2012). Effects of combination of antidiabetic agent and statin on alloxan-induced diabetes with cardiovascular diseases in rats. J Sci Res.

[132] Matafome P, Louro T, Rodrigues L, Crisostomo J, Nunes E, Amaral C, Monteiro P, Cipriano A, Seica R (2011). Metformin and atorvastatin combination further protect the liver in type 2 diabetes with hyperlipidaemia. Diabetes Metab Res Rev.

[133] Singh BK, Singh A, Kumar V (2016). Ameliorative effect of adjunct therapy of metformin with atorvastatin on streptozotocin-induced diabetes mellitus in rats. Drug Res (Stuttg).

[134] Oh JH, Eun Lee J, Jeong Kim Y, Oh TO, Han S, Jeon EK, Shin K, Kim DH, Hye Park C, Lee YJ (2016). Designing of the fixed-dose gastroretentive bilayer tablet for sustained release of metformin and immediate release of atorvastatin. Drug Dev Ind Pharm.

[135] Kandhwal K, Dey S, Nazarudheen S, Arora R, Reyar S, Thudi NR, Monif T, Singh MK, Rao S (2011). Pharmacokinetics of a fixed-dose combination of atorvastatin and metformin extended release versus concurrent administration of individual formulations: a randomized, open-label, two-treatment, two-period, two-sequence, single-dose, crossover, bioequivalence study. Clin Drug Investig.

[136] Scheen AJ (2005). Drug interactions of clinical importance with antihyperglycaemic agents: an update. Drug Saf.

[137] Balasubramanian R, Varadharajan S, Kathale A, Nagraj LM, Periyandavar I, Nayak UP, Sharma A, Bolmall C, Baliga VP (2008). Assessment of the efficacy and tolerability of a fixed dose combination of atorvastatin 10 mg+ metformin SR 500 mg in diabetic dyslipidaemia in adult Indian patients. J Indian Med Assoc.

[138] Krysiak R, Okopien B (2012). Haemostatic effects of metformin in simvastatin-treated volunteers with impaired fasting glucose. Basic Clin Pharmacol Toxicol.

[139] Krysiak R, Okopien B (2012). Lymphocyte-suppressing and systemic anti-inflammatory effects of high-dose metformin in simvastatin-treated patients with impaired fasting glucose. Atherosclerosis.

[140] Krysiak R, Okopien B (2013). The effect of metformin on monocyte secretory function in simvastatin-treated patients with impaired fasting glucose. Metabolism.

[141] Tousoulis D, Koniari K, Antoniades C, Papageorgiou N, Miliou A, Noutsou M, Nikolopoulou A, Marinou K, Stefanadi E, Siasos G (2011). Combined effects of atorvastatin and metformin on glucose-induced variations of inflammatory process in patients with diabetes mellitus. Int J Cardiol.

[142] Tousoulis D, Koniari K, Antoniades C, Miliou A, Noutsou M, Nikolopoulou A, Papageorgiou N, Marinou K, Stefanadi E, Stefanadis C (2010). Impact of 6 weeks of treatment with low-dose metformin and atorvastatin on glucose-induced changes of endothelial function in adults with newly diagnosed type 2 diabetes mellitus: a single-blind study. Clin Ther.

[143] Hao Z, Liu Y, Liao H, Zheng D, Xiao C, Li G (2016). Atorvastatin plus metformin confer additive benefits on subjects with dyslipidemia and overweight/obese via reducing ROCK2 concentration. Exp Clin Endocrinol Diabetes.

[144] Caparros-Martin JA, Lareu RR, Ramsay JP, Peplies J, Reen FJ, Headlam HA, Ward NC, Croft KD, Newsholme P, Hughes JD (2017). Statin therapy causes gut dysbiosis in mice through a PXR-dependent mechanism. Microbiome.

[145] Khan TJ, Ahmed YM, Zamzami MA, Mohamed SA, Khan I, Baothman OAS, Mehanna MG, Yasir M (2018). Effect of atorvastatin on the gut microbiota of high fat diet-induced hypercholesterolemic rats. Sci Rep.

[146] Khan TJ, Ahmed YM, Zamzami MA, Siddiqui AM, Khan I, Baothman OAS, Mehanna MG, Kuerban A, Kaleemuddin M, Yasir M (2018). Atorvastatin treatment modulates the gut microbiota of the hypercholesterolemic patients. OMICS.

[147] Nascimbeni F, Aron-Wisnewsky J, Pais R, Tordjman J, Poitou C, Charlotte F, Bedossa P, Poynard T, Clement K, Ratziu V (2016). Statins, antidiabetic medications and liver histology in patients with diabetes with non-alcoholic fatty liver disease. BMJ Open Gastroenterol.

[148] Ehrmann DA (2005). Polycystic ovary syndrome. N Engl J Med.

[149] Sun J, Yuan Y, Cai R, Sun H, Zhou Y, Wang P, Huang R, Xia W, Wang S (2015). An investigation into the therapeutic effects of statins with metformin on polycystic ovary syndrome: a meta-analysis of randomised controlled trials. BMJ Open.

[150] Chung Y-R, Park SW, Choi S-Y, Kim SW, Moon KY, Kim JH, Lee K (2017). Association of statin use and hypertriglyceridemia with diabetic macular edema in patients with type 2 diabetes and diabetic retinopathy. Cardiovasc Diabetol.

[151] Jorgensen PG, Jensen MT, Biering-Sorensen T, Mogelvang R, Galatius S, Fritz-Hansen T, Rossing P, Vilsboll T, Jensen JS (2016). Cholesterol remnants and triglycerides are associated with decreased myocardial function in patients with type 2 diabetes. Cardiovasc Diabetol.

[152] Hanefeld M, Traylor L, Gao L, Landgraf W (2017). The use of lipid-lowering therapy and effects of antihyperglycaemic therapy on lipids in subjects with type 2 diabetes with or without cardiovascular disease: a pooled analysis of data from eleven randomized trials with insulin glargine 100 U/mL. Cardiovasc Diabetol.

[153] Besseling J, Hutten BA (2016). Is there a link between diabetes and cholesterol metabolism?. Expert Rev Cardiovasc Ther.

[154] Cederberg H, Stancakova A, Yaluri N, Modi S, Kuusisto J, Laakso M (2015). Increased risk of diabetes with statin treatment is associated with impaired insulin sensitivity and insulin secretion: a 6 year follow-up study of the METSIM cohort. Diabetologia.

[155] Chehade JM, Gladysz M, Mooradian AD (2013). Dyslipidemia in type 2 diabetes: prevalence, pathophysiology, and management. Drugs.

[156] Feingold K, Grunfeld C, De Groot LJ, Chrousos G, Dungan K (2000). Role of glucose and lipids in the cardiovascular disease of patients with diabetes. Endotext.

[157] Jaiswal M, Schinske A, Pop-Busui R (2014). Lipids and lipid management in diabetes. Best Pract Res Clin Endocrinol Metab.

[158] Ng DS (2013). Diabetic dyslipidemia: from evolving pathophysiological insight to emerging therapeutic targets. Can J Diabetes.

[159] Soran H, Schofield JD, Adam S, Durrington PN (2016). Diabetic dyslipidaemia. Curr Opin Lipidol.

[160] Schofield JD, Liu Y, Rao-Balakrishna P, Malik RA, Soran H (2016). Diabetes dyslipidemia. Diabetes Ther.

[161] Verges B (2015). Pathophysiology of diabetic dyslipidaemia: where are we?. Diabetologia.

[162] Wang CCL, Hess CN, Hiatt WR, Goldfine AB (2016). Clinical update: cardiovascular disease in diabetes mellitus atherosclerotic cardiovascular disease and heart failure in type 2 diabetes mellitus-mechanisms, management, and clinical considerations. Circulation.

[163] Arca M (2015). Alterations of intestinal lipoprotein metabolism in diabetes mellitus and metabolic syndrome. Atheroscler Suppl.

[164] Tomkin GH, Owens D (2015). Dyslipidaemia of diabetes and the intestine. World J Diabetes.

[165] Arca M, Pigna G, Favoccia C (2012). Mechanisms of diabetic dyslipidemia: relevance for atherogenesis. Curr Vasc Pharmacol.

[166] Perry RJ, Samuel VT, Petersen KF, Shulman GI (2014). The role of hepatic lipids in hepatic insulin resistance and type 2 diabetes. Nature.

[167] Taskinen MR, Boren J (2015). New insights into the pathophysiology of dyslipidemia in type 2 diabetes. Atherosclerosis.

[168] Pagidipati NJ, Pencina M, Sniderman AD (2016). The enigma of glucose and lipid metabolism. JAMA Cardiol.

[169] Parhofer KG (2015). Interaction between glucose and lipid metabolism: more than diabetic dyslipidemia. Diabetes Metab J.

[170] Patel TP, Rawal K, Bagchi AK, Akolkar G, Bernardes N, Dias Dda S, Gupta S, Singal PK (2016). Insulin resistance: an additional risk factor in the pathogenesis of cardiovascular disease in type 2 diabetes. Heart Fail Rev.

[171] Dake AW, Sora ND (2016). Diabetic dyslipidemia review: an update on current concepts and management guidelines of diabetic dyslipidemia. Am J Med Sci.

[172] Halcox J, Misra A (2015). Type 2 diabetes mellitus, metabolic syndrome, and mixed dyslipidemia: how similar, how different, and how to treat?. Metab Syndr Relat Disord.

[173] Paneni F, Cosentino F (2015). Diabetic dyslipidemia. Diabetes and cardiovascular disease.

[174] Szalat A, Durst R, Leitersdorf E (2016). Managing dyslipidaemia in type 2 diabetes mellitus. Best Pract Res Clin Endocrinol Metab.

[175] Ferrannini E, DeFronzo RA (2015). Impact of glucose-lowering drugs on cardiovascular disease in type 2 diabetes. Eur Heart J.

[176] Balakumar P (2014). Implications of fundamental signalling alterations in diabetes mellitus-associated cardiovascular disease. Indian J Biochem Biophys.

[177] Chen SC, Tseng CH (2013). Dyslipidemia, kidney disease, and cardiovascular disease in diabetic patients. Rev Diabet Stud.

[178] Leon BM, Maddox TM (2015). Diabetes and cardiovascular disease: epidemiology, biological mechanisms, treatment recommendations and future research. World J Diabetes.

[179] Paneni F, Beckman JA, Creager MA, Cosentino F (2013). Diabetes and vascular disease: pathophysiology, clinical consequences, and medical therapy: part I. Eur Heart J.

[180] Scheen AJ (2016). Will delayed release metformin provide better management of diabetes type 2?. Expert Opin Pharmacother.

[181] Foster RH, Keam SJ (2006). Metformin extended release. Am J Drug Deliv.

[182] Ali S, Fonseca V (2012). Overview of metformin: special focus on metformin extended release. Expert Opin Pharmacother.

[183] Campbell I, Clarke B, Duncan L (1973). A clinical evaluation of a delayed release preparation of metformin. J Int Med Res.

[184] Wilcock C, Bailey CJ (1994). Accumulation of metformin by tissues of the normal and diabetic mouse. Xenobiotica.

